# The (cost-)effectiveness of exercise therapy adjunct to guideline-concordant care for depression: a pragmatic randomised controlled trial

**DOI:** 10.1192/j.eurpsy.2025.10085

**Published:** 2025-08-15

**Authors:** Michele Schmitter, Ben Wijnen, Daan Creemers, Alice Van Dorp, Peter Oostelbos, Indira Tendolkar, Jasper Smits, Jan Spijker, Janna Vrijsen

**Affiliations:** 1 Depression Expertise Centre, Pro Persona Mental Health Care, Nijmegen, The Netherlands; 2 Behavioural Science Institute, Radboud University Nijmegen, Nijmegen, The Netherlands; 3 Centre for Economic Evaluation, Trimbos Institute (Netherlands Institute of Mental Health and Addiction), Utrecht, The Netherlands; 4 Depression Expertise Centre Youth, GGZ Oost Brabant, Boekel, The Netherlands; 5 GGNet Network for Mental Health Care, Zutphen, The Netherlands; 6 Dutch Depression Association, Amersfoort, The Netherlands; 7 De Hartenboom, Randwijk, The Netherlands; 8 Department of Psychiatry, Donders Institute for Brain, Cognition and Behaviour, Radboud University Medical Centre, Nijmegen, The Netherlands; 9 Department of Psychology & Institute for Mental Health Research, University of Texas at Austin, Austin, TX, USA

**Keywords:** augmentation, cost-effectiveness, depression, exercise, randomised controlled trial

## Abstract

**Background:**

Many patients with major depressive disorder (MDD) do not respond sufficiently to first-line treatments. Due to its biological and psychological mechanisms, exercise may enhance the effectiveness of other MDD treatments. In a pragmatic randomised superiority trial, we evaluated the clinical and cost-effectiveness of exercise therapy adjunct to guideline-concordant care as usual (CAU) for MDD in specialised mental health care.

**Methods:**

MDD outpatients (*N* = 112; Mage = 37; 51% female) were randomized to CAU (96.9% psychotherapy, 59% pharmacotherapy) or CAU + EX (CAU plus 12 weeks of exercise therapy: one supervised and two home-based aerobic sessions/week). Depressive symptoms were assessed using the Inventory of Depressive Symptomatology-Self Report. Remission was evaluated during follow-up by blinded assessors using the Structured Clinical Interview for DSM-5. The economic evaluation followed a societal perspective.

**Results:**

Patients in the CAU + EX condition were significantly more likely than those in CAU to meet the exercise prescription; however, only 22% fully adhered to it. Depressive symptoms decreased from severe to moderate depression in both conditions, with no significant difference between the conditions on symptom reduction (*b* = −0.22, [−0.72, 0.29]) or remission rate (OR = 0.06, [−0.20, 0.32]). Evidence for cost-effectiveness was found in the per-protocol (≥ six supervised exercise sessions) but not in the intention-to-treat sample.

**Conclusions:**

Adjunct exercise therapy does not provide additional clinical benefits or cost-effectiveness in specialized mental health care. Low adherence to the exercise prescription limits its potential. Cost-effectiveness may be achievable with higher adherence, warranting emphasis on strategies to improve adherence in this population.

## Introduction

Major depressive disorder (MDD) is prevalent, disabling, and costly [1, 2]. First-line treatments, including pharmacotherapy and psychotherapy, are effective for only 50–60% of patients [3–5], and recurrence is common, with 60% relapsing within five years [6]. Additionally, neither pharmacotherapy nor psychotherapy directly improves physical health, despite MDD’s association with increased somatic morbidity [7, 8], and a reduced life expectancy of nearly 15 years [9]. This underscores the need for more holistic treatment approaches.

As a structured and supervised monotherapy, exercise is as effective as psychotherapy and pharmacotherapy in reducing depressive symptoms [10]. The evidence-based prescription for MDD consists of three weekly moderate-intensity 45–60-minute aerobic exercise sessions (of which at least one should be professionally supervised) provided over 10–14 weeks [11, 12]. Exercise improves cognition and quality of life [13], as well as physical health [14]. Furthermore, exercise enhances memory and learning [15], likely by promoting neuroplasticity, and positively influences neurotransmitters such as serotonin [16, 17]. It may therefore complement the core mechanisms of pharmacotherapy and psychotherapy [18–20]. This makes it a promising adjunct to MDD treatments. Indeed, multiple trials have demonstrated that exercise enhances the effects of pharmacotherapy [21–23], cognitive behavioural therapy (CBT) [24] and their combination [25]. This evidence is compelling; however, robust data on long-term effects and cost-effectiveness in routine practice are lacking and urgently needed for the effective implementation of exercise therapy in specialized care.

Therefore, we investigated the clinical and cost-effectiveness of guideline-concordant care as usual (CAU) for MDD outpatients with or without the addition of evidence-based exercise therapy (CAU + EX) in a pragmatic randomised controlled trial [26]. We hypothesised that CAU + EX would be superior to CAU in reducing depressive symptoms, achieving remission, and improving relevant secondary outcomes (i.e., disability, motivation and energy, rumination, self-esteem, negative memory bias, and physical fitness).

## Method

### Trial design

In this pragmatic multicentre RCT, patients with MDD from four Dutch specialized mental health care centres were randomized to CAU or CAU + EX, with assessments up to 15 months post-baseline. Assessments were conducted online at baseline (i.e., before the start of treatment or before the third treatment week, the latest; T0), and at 3 (T1), 6, (T2), and 9 (T3) weeks during treatment. Post-treatment assessments were conducted online (i.e., questionnaires) and via telephone (i.e., diagnostic interviews to assess remission) at 12 weeks (T4), and during follow-up at 6 (T5), 9 (T6), 12 (T7), and 15 (T8) months. After trial completion, the CAU condition was offered exercise therapy. Methods are detailed in the published protocol paper [26] and summarised below. All procedures complied with the Helsinki Declaration (2013), were approved by the CMO Arnhem-Nijmegen ethical review board (NL72080.091.19), and the trial was registered at the International Clinical Trial Registry Platform (NL8432). We followed CONSORT [27], and Dutch and Cheers guidelines for the economic evaluation [28, 29].

### Sample size

Before the trial, we calculated a required sample size of *N* = 120 (*α* = .05, power (1−*β*) = 0.80, two-tailed test), based on an expected effect size of *g* ≥ 0.70, derived from a meta-analysis of similar studies [26]. After consultation with the grant provider, we conducted an interim analysis on available T1 (*n* = 56) and T4 (*n* = 52) data. This analysis accounted for the actual pre- and post-treatment correlation of .64 of the main outcome (i.e., depressive symptom severity measured with the Inventory of Depressive Symptomatology-Self Report; IDS-SR), an ICC of .058 (T1) and .107 (T4) (i.e., a smaller design effect than originally expected), and a mean cluster size of three patients per clinician, resulting in a recalibrated sample size of *N* = 54. To account for 30% dropout based on the rate at the time of the interim analysis, we needed to recruit a minimum of *N* = 78 (*n* = 39 per condition) and eventually recruited *N* = 112 (*n* = 57 CAU + EX; *n* = 55 CAU).

### Participants

The trial population consisted of patients (> 16 years) with MDD according to the Diagnostic and Statistical Manual of Mental Disorders [30]. Exclusion criteria included: a lifetime history of manic episodes; current psychosis; persistent depression (i.e., the current depressive episode lasting 2 years or longer) or dysthymic disorder; high health risks associated with physical activity, as per the Physical Activity Readiness Questionnaire (PAR-Q) [31], insufficient comprehension of Dutch; physical, cognitive, or intellectual impairments interfering with participation or informed consent; and receiving more than three weeks of CAU before inclusion.

### Randomisation and masking

Randomisation was stratified by sex and treatment centre using Castor EDC, which enrolled patients consecutively based on a four, six, or eight block design. Separate blocks were created for each stratum, with a new block randomly generated after the previous one was filled. Patients were randomly allocated within a block. The trained researchers assessing remission from MDD post-exercise-treatment and during follow-up, as well as those performing the statistical analyses, were blinded.

### Procedures

Clinicians referred eligible patients to the researchers between March 2020 and January 2023, with the first inclusion on 10 March 2020. Interested patients received information about study participation during a phone call, and an information letter via email, followed by a minimum 48-hour reconsideration period before providing (written) informed consent. Due to local restrictions on in-person interactions, patients were randomised after providing verbal consent via phone at several periods during the COVID-19 pandemic.

### Treatment

#### CAU

The CAU treatment followed the Dutch multidisciplinary guidelines [32] for managing MDD, incorporating pharmacological (CAU: 61% and CAU + EX: 57% at baseline) and/or psychological treatments (CAU: 97% and CAU + EX: 96% at baseline) provided individually or in groups (see Supplementary Material 1 [SM] for details). By the end of the trial (i.e., 15 months post-baseline), most patients (still) received treatment for MDD (CAU: 67%; CAU + EX: 64%). CAU patients were permitted to receive individual psychomotor therapy and engage in self-directed exercise but did not receive structured exercise therapy.

#### Adjunct exercise

The 12-week evidence-based exercise therapy for MDD [11, 12] consisted of three weekly moderate-intensity aerobic exercise sessions, each lasting 45 min. Patients exercised once a week under the supervision of a psychomotor therapist or trained nurse at the treatment centre, and were committed to exercising twice a week at home. At-home exercise at the required intensity was prescribed using the evidence-based *Exercise and Depression Toolkit* [33], adapted for the Dutch mental health care setting. It includes behavioural techniques to promote adherence, such as goal setting, scheduling, psychoeducation on mental health benefits, and mood tracking. The toolkit was introduced in the first supervised session.

Supervised sessions typically involved group-based running or indoor cycling (spinning), but other forms of exercise were occasionally offered to accommodate patients’ abilities and preferences. Moderate intensity was defined as 64–76% of HRmax (220 – age) and self-monitored during the sessions with the aid of a non-invasive activity tracker (Fitbit). Patients completed a brief survey at each supervised exercise session, reporting the intensity, duration, and frequency of their weekly exercise, along with any direct mood benefits. At the end of each session, they also shared their exercise experience, including the struggles and benefits, and planned the upcoming week’s exercise with the therapist.

### Outcomes

#### Depressive Symptoms

Severity of depressive symptoms was assessed using the Dutch version of the 30-item IDS-SR (T0-T8) [34]. This scale measures depressive symptoms on a four-point Likert scale, with higher scores indicating greater severity. Cronbach’s alpha was .847 at baseline, indicating high internal consistency. Norms are: 14–25 (mild), 26–38 (moderate), 39–48 (severe), and 49 and above (very severe) [35].

#### Remission

The Structured Clinical Interview for DSM-5 (SCID-5-S) [36] was used to assess MDD remission post-exercise-therapy (T4) and during follow-up assessments (T5-T8), defined as less than five depressive symptoms in the past two weeks.

#### Exercise and physical activity

To assess physical activity levels, including exercise, the Dutch version of the International Physical Activity Questionnaire (IPAQ) was used (T0-T8), which measures physical activity across different domains (e.g., leisure-time or work-related). Exercise therapy was aimed at increasing frequency and duration of leisure-time exercise; hence we used the minutes spent on leisure-time moderate and vigorous-intensity exercise in the analyses of exercise adherence. Overall physical activity levels were analysed as secondary outcomes (Supplementary Material 9).

#### Health-related quality of life

As recommended by Dutch guidelines for cost-effectiveness studies [29], quality of life (assessed with the EuroQol 5-dimensions 5-level [EQ-5D-5L] and the Dutch tariff) [37] was used as an outcome measure for the cost-utility analysis. Patients rated their health across five domains (i.e., mobility, self-care, usual activities, pain/discomfort, and anxiety/depression), which were converted into a utility score in which 0 represents dead and 1 perfect health (note: for severe health states a utility below 0 is possible, indicating a state worse than dead). Utilities were used to calculate quality-adjusted life years (QALYs) post-treatment and over the follow-up period by weighing the time spent in each health state (i.e., linear interpolation).

#### Cost measures

The Trimbos/iMTA questionnaire for psychiatric illness costs (TiC-P) assessed societal costs of psychiatric treatment [38]. Costs were divided into health care, patient and family, and productivity losses (i.e., productivity losses for (un)paid work, including absenteeism and presentism). After cleaning the data (e.g., hours of (un)paid work set to a maximum of 40 h/week and the maximum GP visits were set to five/week), total costs in euros were calculated by multiplying resource use with unit costs and summing them [39]. Health care costs followed Dutch guidelines [29] with commercial prices used when guidelines were lacking. Pharmaceutical costs were based on daily defined doses in line with Dutch guidelines [29], “Medicijnkosten.nl”). Travel costs were estimated from the mean distance to health care providers per Dutch costing guidelines [29]. Productivity losses were calculated with the friction cost method (136 days), adjusted to 2022 prices, with no discounting applied for the 15-month study period.

#### Statistical approach

Analyses were conducted using R 4.1.2, following the intention-to-treat principle. Descriptive statistics were calculated, followed by mixed model analyses using the *lme4* package [40]. We assessed exercise adherence by classifying patients based on whether they met the 135-minute exercise prescription, using a logistic mixed model with condition as a predictor and a random effect for patients. To evaluate the adjunct exercise therapy’s effectiveness, missing data were multiple imputed (*N* = 5), using predictive mean matching via the Multivariate Imputation by Chained Equations (MICE) package [41], and a linear mixed model assessed the condition-by-time interaction for depressive symptoms, accounting for time [42], with a random effect for patients clustered within treatment centres. Results were pooled using Rubin’s rule [43]. For remission, missing data were (conservatively) treated as no remission and analysed using a logistic mixed model with condition-by-time interaction, condition, and time as predictors, and a random effect for patients.

In the economic evaluation, we performed seemingly unrelated regression equations (SURE) to simultaneously analyse costs and outcomes, while accounting for baseline utility or baseline costs. Since costs are usually non-normally distributed, the SURE models were bootstrapped (5000 times). Missing data were similarly imputed based on predictive mean matching but nested in the nonparametric bootstraps of the SURE models using single imputation for each bootstrap replication [44]. In all imputations (i.e., multiple imputation in effect analysis and single imputation nested in bootstraps in the economic evaluation), we used baseline variables that were predictive of depressive symptoms, costs, or missingness, to impute the missing values. The incremental cost-utility ratio (ICUR) was determined using the differences in costs and QALYs between CAU and CAU + EX. Bootstrapped ICERs/ICURs were plotted on a cost-effectiveness plane, and a cost-effectiveness acceptability curve (CEAC) was generated to assess the likelihood of the exercise treatment being cost-effective at various willingness-to-pay (WTP) values per QALY. For this trial, a WTP threshold of €50,000 per QALY was assumed for moderate to severe depression severity [45]. A societal perspective was adopted for the cost-utility analysis, incorporating all direct and indirect costs associated with the intervention. Sensitivity analyses, including those from a healthcare perspective, are provided in the Supplementary Material. Additionally, treatment effectiveness and economic evaluations were also conducted on a per-protocol sample, defined as patients from CAU + EX condition completing at least six supervised exercise sessions, with those attending fewer sessions reassigned to the CAU condition for these analyses.

## Results

**Figure 1.**
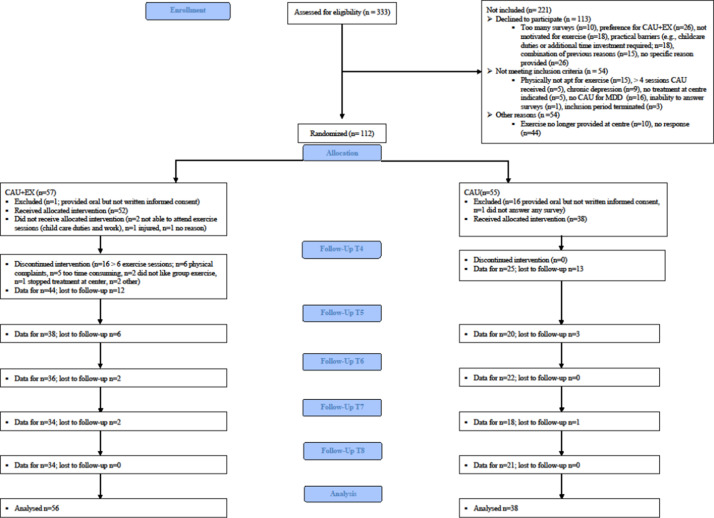
CONSORT flow diagram. *Note:* Two researchers independently assessed the reasons for excluding patients from the trial. Inter-rater agreement was excellent for the exclusion categories (*k* = 1), indicating perfect agreement. Additionally, the reasons provided by the patients were also evaluated independently by two researchers, showing substantial agreement (*k* = .802). Agreement between the raters was reached after discussion and resolution of discrepancies.

Baseline characteristics are summarised in Table 1. Figure 1 displays the patient flow. Discontinuation rates after randomisation differed significantly between conditions, with more patients withdrawing in the CAU condition (31%) than in the CAU + EX condition (2%), *χ*^2^(1) = 16.14, *p* < .001, OR = 0.04, 95% CI [0.01, 0.30]. CAU patients cited not receiving exercise therapy as the primary reason for withdrawing.

**Table 1.** Baseline characteristics

	Total sample (*N* = 94)	CAU + EX (*N* = 56)	CAU (*N* = 38)
Sex, male	46 (48.9%)	28 (50.0%)	18 (47.4%)
Age in years, mean (SD)	36.6 (12.7)	36.9 (13.1)	36.2 (12.2)
Nationality			
Dutch	84 (89.4%)	47 (83.9%)	37 (97.4%)
Other (Former soviet union, former Yugoslavia, Iran, Germany, Austria, El Salvador, Israel, Turkey, Brazil, Romania)	10 (10.6%)	9 (16.1%)	1 (2.6%)
Marital status			
Married/cohabiting	38 (40.4%)	22 (39.3%)	16 (42.1%)
Unmarried/divorced/widowed	56 (59.6%)	34 (60.7%)	22 (57.9%)
Living situation			
Living alone	31 (33.0%)	18 (32.1%)	13 (34.2%)
Living together (with partner, family, or group)	63 (67.0%)	38 (67.9%)	25 (65.8%)
Education level			
Low	19 (20.2%)	9 (16.1%)	10 (26.3%)
Moderate	45 (47.9%)	27 (48.2%)	18 (47.4%)
High	30 (31.9%)	20 (35.7%)	10 (26.3%)
Employment status			
Full time	16 (17.0%)	10 (17.9%)	6 (15.8%)
Student	15 (16.0%)	9 (16.1%)	6 (15.8%)
Part-time (6–32 h/week)	21 (22.3%)	13 (23.2%)	8 (21.1%)
Not working (e.g., unemployed, sick leave, homemaker)	42 (44.7%)	24 (42.9%)	18 (47.4%)
Comorbid psychological diagnoses	41 (43.6%)	24 (42.9%)	17 (44.7%)
Anxiety disorder	19 (45.6%)	12 (50%)	7 (41.2%)
Posttraumatic stress disorder	3 (8.9%)	0 (0%)	3 (17.7%)
Personality disorder	6 (15.1%)	3 (12.5%)	3 (17.7%)
Developmental disorder	13 (31.4%)	8 (33.3%)	5 (29.4%)
Substance use disorder	6 (15.1%)	3 (12.5%)	3 (17.7%)
Other	5 (12.2%)	3 (12.5%)	2 (11.8%)
Somatic disorder (rheumatic disorder, cardiovascular disease, lung diseases, cancer, gastrointestinal disease)	24 (25.5%)	16 (28.6%)	8 (21.1%)
VO_2_max at baseline, Mean (SD)	31.62 (10.41)	35.48 (6.34)	27.76 (14.48)

*Note:* Age range was 18–65 years. VO_2_max was used as index of physical fitness. The fitness test was completed in a small subsample of *n* = 13 from CAU + EX and *n* = 7 from the CAU condition. Considering the sample’s mean age, both conditions show below-average physical fitness.

### Exercise adherence

Patients in the CAU + EX condition attended 72% of supervised exercise sessions (*M* = 9.27) and 50% of home-based sessions (*M* = 13.04), 73% of which were at moderate or higher intensity. This included four patients who did not start exercise therapy. The per-protocol CAU + EX sample (≥ 6 supervised sessions; *n* = 40, 71%) attended 92% of supervised sessions (*M* = 11.08) and 67% of home-based sessions (*M* = 16.48), 72% of which were at moderate or higher intensity. There were no significant differences in baseline characteristics between patients in the per-protocol sample and patients who attended fewer than six supervised exercise sessions (Supplementary Material 2). The logistic mixed model analysis revealed that patients in the CAU + EX condition were significantly more likely to meet the exercise prescription of 135 min/week moderate to higher-intensity exercise both during the treatment phase (T0-T4), OR = 2.05, 95% CI [0.54, 3.57], *p* = .008, and during follow-up (T5–T8), OR = 1.23, 95% CI [0.14, 2.33], *p* = .028, compared to CAU. Patients in the CAU + EX condition exercised more minutes per week during treatment (CAU + EX: *M* = 98 min; CAU: *M* = 36 min), and follow-up (CAU + EX: *M* = 100 min; CAU: *M* = 48 min; Supplementary Material 3 for details). In the CAU + EX condition, 21% of patients adhered to the prescription during the treatment phase, and 19% during follow-up. Trends in home-based sessions and mood benefits are detailed in Supplementary Material (Supplementary Material 3).

### Treatment effectiveness

There was a significant effect of time on depressive symptoms, *b* = −1.02, 95% CI [−1.56, −0.47], *p* = .001. However, the time-by-condition interaction was not significant (Table 2), indicating both conditions improved similarly in depressive symptoms over time, with an average decrease from severe to moderate levels. Also, when taking remission rates as outcome, the effect of time was significant, OR = 0.73, 95% CI [0.53, 0.94], *p* > .001, but not the time-by-condition interaction (Table 2).

**Table 2.** Depressive symptoms, remission, utility, and costs per condition over time and treatment effects

Measure	T0	T4	T5	T6	T7	T8	Cumulative costs (95% CI)	Treatment effect (95% CI)
**Depressive symptoms, mean (SD)**							*b* = −0.22, [−0.72, 0.29], *p* = .396	
CAU + EX	42.32 (11.00)	34.25 (15.19)	32.68 (14.63)	30.47 (12.36)	29.26 (13.15)	27.70 (14.03)		
CAU	39.92 (12.26)	30.04 (13.17)	28.55 (15.28)	26.86 (15.57)	27.94 (15.26)	26.66 (15.74)		
**Remission (%)**							*OR* = 0.06, [−0.20, 0.32], *p* = .662	
CAU + EX		9 (19.14)	14 (53.57)	17 (52.38)	10 (52.63)	19 (51.35)		
CAU		9 (36.00)	15 (31.82)	11 (45.95)	16 (44.44)	11 (50.00)		
**Utility, mean (SD)**								
CAU + EX	0.49 (0.22)	0.57 (0.24)	0.64 (0.23)	0.64 (0.27)	0.66 (0.24)	0.67 (0.25)		
CAU	0.49 (0.24)	0.60 (0.26)	0.61 (0.28)	0.63 (0.23)	0.63 (0.21)	0.64 (0.31)		
**Health care costs, mean (SD)**								
CAU + EX	1050 (958)	1040 (944)	872 (758)	629 (597)	552 (509)	660 (724)	11901 (9740–14402)	
CAU	1040 (959)	1050 (718)	543 (376)	727 (528)	632 (537)	821 (858)	11522 (8953–14255)	
**Patient and family costs, mean (SD)**								
CAU + EX	213 (318)	209 (501)	226 (479)	148 (308)	114 (250)	94.2 (159)	2756 (1819–3912)	
CAU	287 (534)	173 (247)	105 (155)	272 (629)	115 (202)	237 (597)	2965 (1479–5144)	
**Productivity losses, mean (SD)**								
CAU + EX	1270 (2540)	774 (2320)	978 (2670)	364 (682)	163 (371)	207 (322)	9019 (5256–13520)	
CAU	1570 (3180)	348 (870)	202 (540)	106 (192)	210 (476)	466 (1490)	5879 (2929–9822)	
**Total societal costs, mean (SD)**								See Figure 2
CAU + EX	2530 (2820)	2020 (2560)	2080 (2830)	1140 (1340)	830 (773)	1220 (1680)	23677 (18971–28910)	
CAU	2900 (3470)	1570 (1230)	850 (674)	1100 (842)	956 (697)	1270 (1490)	20366 (16000–25540)	

*Note:* Depressive symptoms were assessed with the IDS-SR and the SCID was used to assess the absence of a MDD classification indicative of remission. The cumulative costs display the imputed costs and bootstrapped confidence intervals. All other costs display raw (non-imputed) data. For the treatment effect, we report the results for the time-by-condition interaction with depressive symptoms and remission as outcome. The estimates, 95% confidence intervals and *p* values are derived from the mixed model analyses with imputed data.

Results were similar in the per-protocol sample (CAU + EX: *n* = 40; CAU: *n* = 54). For depressive symptoms the effect of time was significant, *b* = −1.02, 95% CI [−1.58, −0.47], *p* = .002, but not the time-by-condition interaction, *b* = −0.27, 95% CI [−0.84, 0.29], *p* = .346, and also for remission the effect of time was significant, OR = 0.81, 95% CI [0.61, 1.00], *p* > .001, but not the time-by-condition interaction, OR = −0.10, 95% CI [−0.37, 0.17], *p* = .476. Results showed a similar pattern in the non-imputed analyses (Supplementary Material 4; which includes a plot illustrating individual differences in treatment response), when accounting for possible differences in treatment delivery during the COVID-19 pandemic (Supplementary Material 5), and accounting for baseline exercise (Supplementary Material 6), for IDS-SR subscales as outcome (Supplementary Material 7), the cumulative effect of weekly exercise sessions as predictor (Supplementary Material 8), exploratory moderation and responder prediction analyses (Supplementary Material 9) and for the secondary outcomes (Supplementary Material 10).

### Economic evaluation

**Figure 2.**
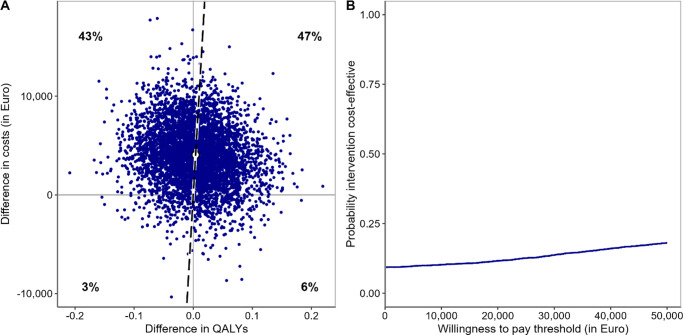
The cost-effectiveness plane (A) and cost-utility acceptability curve (B) for the economic evaluation of adjunct exercise. *Note:* The cost-effectiveness plane (A) illustrates the incremental costs and effects of CAU + EX compared to CAU. The dashed line represents the mean incremental cost-effectiveness ratio (ICER) for the comparison. The cost-utility acceptability curve (B) shows the probability that the exercise treatment is cost-effective across a range of WTP thresholds, up to €50,000 per additional QALY gain.

The cost-utility analysis based on bootstrapped data showed a non-significant QALY difference of 0.004, 95% CI [−0.09, 0.11], in favour of CAU + EX, with CAU + EX incurring an additional cost of €4,054, 95% CI [−1998, 10093]. This resulted in ICUR of €1,018,771, which exceeds the WTP threshold of €50,000. The cost-effectiveness plane and CEAC are shown in Figure 2, with the CEAC indicating an 18% probability of CAU + EX being cost-effective at a WTP threshold of €50,000. Similar results were observed in the sensitivity analyses (Supplementary Material 11). From a health care perspective, CAU + EX was neither deemed cost-effective, with a QALY difference of 0.004, 95% CI [−0.09, 0.11], in favour of CAU + EX, and additional costs of €438, 95% CI [−3,030.17, 3,968.81]. This resulted in an ICUR of €109,953, again exceeding the WTP threshold of €50,000 (see Supplementary Material 11 for the cost-effectiveness plan and CEAC).

In the per-protocol sample, we found a non-significant QALY difference of 0.044, 95% CI [−0.06; 0.14], in favour of CAU + EX, with an additional cost of only €976, 95% CI [−5389, 7664], leading to an ICUR of €22,269. The CEAC in this case showed that the probability of CAU + EX being cost-effective at a WTP threshold of €50,000 was 61% (see Supplementary Material 12 for cost-effectiveness plane and CEAC). Mean costs, utility, depressive symptoms, and number of patients in remission per measurement occasion are presented in Table 2 (further details Supplementary Material 13).

### Adverse events

One serious adverse event occurred: a CAU patient committed suicide, deemed unrelated to the trial interventions by the treating psychiatrist.

## Discussion

We evaluated the clinical and cost-effectiveness of evidence-based exercise therapy as an adjunct to guideline-concordant care for MDD outpatients in specialised mental health care. No evidence supported the superiority of adjunct exercise therapy in reducing depressive symptoms or achieving remission. Exercise therapy was cost-effective only for patients who attended at least six supervised sessions in the per-protocol sample.

These results contrast with meta-analytic findings [46], which support the efficacy of exercise augmentation. Unlike other clinical studies [21, 22, 24], our trial involved patients from routine specialized care with severe depression, comorbidity, and physical health impairments. Furthermore, 60% received combined treatments, and medication adjustments were allowed, reflecting real-world practice [47], which may explain the null findings.

Critical factors that may have influenced our results are exercise dose and adherence. It is possible that the exercise dose prescribed in our study was too low to yield clinical effects in this disabled sample. There is some evidence suggesting that exercise may be most effective when the prescribed dose is high-intensity exercise [23, 48]. Dose-ranging studies are important to move the literature forward. Especially when considering high-intensity prescriptions, personalising exercise programs, allowing patients to select activities based on their abilities and preferences [49, 50], and offering more supervision [46] may be required for adherence and tolerability. Additionally, offering exercise therapy prior to CBT sessions may help directly address exercise barriers [51], negative thoughts related to exercise [52], and logistical challenges such as extra travel time. This approach could leverage both the immediate and cumulative effects of exercise, potentially improving adherence and enhancing symptom reduction.

Interestingly, the augmentation was cost-effective for patients who attended six or more supervised exercise sessions. Though the QALY difference between the per-protocol sample and CAU was small and not significant, it is considered clinically meaningful [53]. The most plausible explanation for this finding is that adjunct exercise may have impacted outcomes not assessed in this trial, such as somatic health. Previous studies suggest that as few as six exercise sessions can significantly improve somatic health in MDD patients [54, 55], which might explain the observed cost-effectiveness through potential reductions in health care utilization or related costs. However, these results should be interpreted with caution, as the per-protocol analysis was no longer based on a randomised sample. While no baseline differences were found between the per-protocol sample and those who dropped out after fewer than six sessions, it is possible that healthier patients incurred lower costs. Future studies should structurally assess physical health outcomes in patients with severe depression levels, as they are equally important as depression outcomes in this impaired population [9].

### Strengths and limitations

The trial’s pragmatic design is a key strength, offering high ecological validity. However, varying psychological and pharmacological interventions in the CAU treatment may have obscured exercise effects. The trial was also affected by COVID-19, leading to missing data from telephone interviews (28% on average) and reduced statistical power for remission. Similarly, COVID-19 restrictions limited fitness test completion, resulting in insufficient data for conclusions. Additionally, selection bias may have occurred as patients could not be blinded to their treatment, likely contributing to higher discontinuation rates in the CAU condition. At the same time, this also underscores the appeal of exercise as an adjunct therapy for patients with MDD. Finally, because this sample consisted of patients with severe depression and high rates of comorbidity in specialized care, the results may not generalize to less severely affected patients or those treated in community or primary care settings, where adjunct exercise therapy might augment usual care. In line with this, our exploratory responder analyses suggest that certain subgroups—including patients with lower baseline disability, fewer somatic comorbidities, and less severe depressive symptoms—may be more likely to benefit. Although these trends were not statistically significant and should be interpreted cautiously, they may help generate hypotheses for future research on individual predictors of response. We also found some indication that older patients and women might be less likely to respond to adjunct exercise. Sex has previously been discussed as a potential moderator [56, 57], the roles of both age and sex warrant further investigation. In contrast, patients with comorbid diagnoses may be more likely to respond, which also warrants further study.

Future studies should prioritize improving adherence through personalisation and supervision to reduce dropout rates in patients in specialized care, as our cost-effectiveness findings suggest that benefits depend on attending multiple sessions. Furthermore, even with adequate adherence, the prescribed dose and intensity might require adjustment—such as higher intensity or longer duration—to achieve clinical improvements in this complex patient population. Personalised exercise prescriptions—tailored to individual health profiles and preferences—and offering alternative modalities such as yoga or resistance training [58], combined with more supervision and support, may improve feasibility, adherence, and clinical benefit.

## Conclusion

Adjunct evidence-based exercise therapy offers no additional clinical benefits and is not cost-effective in reducing depressive symptoms or achieving remission for MDD outpatients in specialized mental health care. Therefore, the results do not support widespread implementation of the exercise prescription (i.e., one supervised and two home-based moderate-intensity 45-minute sessions per week [11]), as an adjunct treatment for MDD. However, when patients attend six or more supervised exercise sessions, adjunct exercise therapy may become cost-effective. This warrants further research to improve treatment adherence in this impaired population and to identify which patients would benefit most from exercise therapy.

## Supporting information

10.1192/j.eurpsy.2025.10085.sm001 Schmitter et al. supplementary material Schmitter et al. supplementary material

## Data Availability

The data and R code that support the findings of this study are archived in the Radboud University Data Repository (https://doi.org/10.34973/8v18-zy08) and are available from the corresponding author upon reasonable request. Additionally, to promote open science and facilitate early dissemination, this manuscript has been made available as a preprint on the Open Science Framework prior to peer review and formal publication. The preprint can be accessed at: https://doi.org/10.34973/yfdw-m947.

## Supplementary material

The supplementary material for this article can be found at http://doi.org/10.1192/j.eurpsy.2025.10085.
