# Supplementary material for: The (cost-)effectiveness of exercise therapy adjunct to guideline-concordant care for depression: a pragmatic randomised controlled trial
Source: Eur Psychiatry. 2025 Aug 15;68(1):e124. doi: 10.1192/j.eurpsy.2025.10085 (PMC12438981; doi:10.1192/j.eurpsy.2025.10085)
Supplement: Schmitter et al. supplementary material [file S0924933825100850sup001.docx]

**The (cost-)effectiveness of exercise therapy adjunct to guideline-concordant care for depression: A pragmatic randomised controlled trial**

**Supplementary material**

**SM1: Further information on CAU in both conditions**

Table 1 summarizes the number of patients per condition using benzodiazepines and different categories of antidepressant medication across measurement points (i.e., T0-T8). Additionally, it provides data on psychological treatment at baseline.

Table 1

*Further information on CAU throughout the study in both conditions*

|  | **Time** | **Treatment** | **CAU+EX (N = 56)** | **CAU (N = 38)** |
| --- | --- | --- | --- | --- |
|  | **T0** |  |  |  |
|  |  | **Psychological treatment** | 54 (96.4%) | 37 (97.4%) |
|  |  | Cognitive Behavioural Therapy | 35 (62.5%) | 23 (60.5%) |
|  |  | Interpersonal therapy | 2 (3.6%) | 1 (2.6%) |
|  |  | Psychodynamic therapy | 1 (1.8%) | 1 (2.6%) |
|  |  | Behaviour therapy | 3 (5.4%) | 4 10.5%) |
|  |  | Day-hospital | 12 (21.4%) | 8 (21.1%) |
|  |  | Other | 3 (5.4%) | 6 (15.8%) |
|  |  | **Benzodiazepines** | 18 (32.1%) | 11 (28.9%) |
|  |  | **Antidepressants** | 33 (58.9%) | 23 (60.5%) |
|  |  | SSRI | 19 (33.9%) | 10 (26.3%) |
|  |  | TCA / SNRI / other AD | 8 (14.3%) | 8 (21.1%) |
|  |  | 2 AD / 1 AD+AP | 5 (8.9%) | 5 (13.2%) |
|  | **T4** |  |  |  |
|  |  | **Benzodiazepines** | 26 (46.4%) | 7 (18.4%) |
|  |  | **Antidepressants** | 32 (57.1%) | 16 (42.1%) |
|  |  | SSRI | 14 (25.0%) | 4 (10.5%) |
|  |  | TCA / SNRI / other AD | 12 (21.4%) | 9 (23.7%) |
|  |  | 2 AD / 1 AD+AP | 6 (10.7%) | 3 (7.9%) |
|  | **T5** |  |  |  |
|  |  | **Benzodiazepines** | 16 (28.6%) | 14 (36.8%) |
|  |  | **Antidepressants** | 30 (53.6%) | 20 (52.6%) |
|  |  | SSRI | 12 (21.4%) | 5 (13.2%) |
|  |  | TCA / SNRI / other AD | 11 (19.6%) | 12 (31.6%) |
|  |  | 2 AD / 1 AD+AP | 5 (8.9%) | 4 (10.5%) |
|  | **T6** |  |  |  |
|  |  | **Benzodiazepines** | 11 (19.6%) | 11 (28.9%) |
|  |  | **Antidepressants** | 20 (35.7%) | 15 (39.5%) |
|  |  | SSRI | 11 (19.6%) | 2 (5.3%) |
|  |  | TCA / SNRI / other AD | 5 (8.9%) | 11 (28.9%) |
|  |  | 2 AD / 1 AD+AP | 3 (5.4%) | 3 (7.9%) |
|  | **T7** |  |  |  |
|  |  | **Benzodiazepines** | 9 (16.1%) | 12 (31.6%) |
|  |  | **Antidepressants** | 21 (37.5%) | 12 (31.6%) |
|  |  | SSRI | 8 (14.3%) | 1 (2.6%) |
|  |  | TCA / SNRI / other AD | 8 (14.3%) | 9 (23.7%) |
|  |  | 2 AD / 1 AD+AP | 2 (3.6%) | 1 (2.6%) |
|  |  | MAOI | 1 (1.8%) | 0 (0%) |
|  | **T8** |  |  |  |
|  |  | **Benzodiazepines** | 11 (19.6%) | 13 (34.2%) |
|  |  | **Antidepressants** | 18 (32.1%) | 14 (36.8%) |
|  |  | SSRI | 5 (8.9%) | 1 (2.6%) |
|  |  | TCA / SNRI / other AD | 8 (14.3%) | 12 (31.6%) |
|  |  | 2 AD / 1 AD+AP | 2 (3.6%) | 1 (2.6%) |
|  |  | MAOI | 1 (1.8%) | 0 (0%) |

***Note.*** The time column indicates the time of the assessment, T0 at baseline, T4 post-exercise-treatment (i.e., 12 weeks), and during follow-up with T5 after 3 months post-treatment, T6 after 6 months post-treatment, T7 after 9 months post-treatment and T8 after 12 months post-treatment. Other psychological treatment at baseline included mindfulness-based cognitive therapy, schema therapy and psychomotor therapy. SSRI refers to selective serotonin reuptake inhibitors, TCA refers to tricyclic antidepressants, SNRI refers to [serotonin–norepinephrine reuptake inhibitor, AD refers to antidepressant, AP refers to antipsychotic medication, and MAOI refers to](https://en.wikipedia.org/wiki/Serotonin%E2%80%93norepinephrine_reuptake_inhibitor) [monoamine oxidase inhibitor.](https://en.wikipedia.org/wiki/Monoamine_oxidase_inhibitor)

**SM2: Differences between exercise dropouts with less than six exercise sessions and the per-protocol sample**

Table 2 compares patients who attended less than six supervised exercise sessions (i.e., dropouts from the exercise treatment) with those in the per-protocol sample (i.e., attended at least six supervised exercise session), using chi-square tests for categorical variables and *t*-tests for continuous variables. Overall, no significant differences were found between the two groups.

Table 2

*Differences between the exercise dropouts with less than six sessions and per-protocol sample*

|  | **Dropouts (N = 16)** | **Per-protocol (N = 40)** | ***p*** |
| --- | --- | --- | --- |
| **Gender** |  |  |  |
| Male | 7 (43.8%) | 21 (52.5%) | 0.767 |
| **Age in years, Mean (SD)** | 34.6 (13.3) | 37.8 (13.1) | 0.429 |
| **Nationality** |  |  |  |
| Dutch | 15 (93.8%) | 32 (80.0%) | 0.388 |
| Other | 1 (6.3%) | 8 (20.0%) |  |
| **Marital status** |  |  |  |
| Married/Cohabiting | 5 (31.3%) | 17 (42.5%) | 0.634 |
| Unmarried/Divorced/Widowed | 11 (68.8%) | 23 (57.5%) |  |
| **Living situation** |  |  |  |
| Living alone | 4 (25.0%) | 14 (35.0%) | 0.684 |
| Living together (i.e., with partner, family or community living | 12 (75.0%) | 26 (65.0%) |  |
| **Education level** |  |  |  |
| Low | 2 (12.5%) | 7 (17.5%) | 0.713 |
| Moderate | 7 (43.8%) | 20 (50.0%) |  |
| High | 7 (43.8%) | 13 (32.5%) |  |
| **Employment status** |  |  |  |
| Full time working | 3 (18.8%) | 7 (17.5%) | 0.333 |
| Student | 4 (25.0%) | 5 (12.5%) |  |
| Part-time working (6-32h) | 5 (31.3%) | 8 (20.0%) |  |
| Not working (e.g., unemployed, sick leave or homemaker) | 4 (25.0%) | 20 (50.0%) |  |
| **Comorbid psychological diagnoses** | 7 (43.8%) | 17 (42.5%) | 0.624 |
| **Somatic disorder** | 5 (31.3%) | 11 (27.5%) | 1.000 |
| **Depressive symptoms (IDS-SR)** | 41.3 (13.7) | 42.8 (9.89) | 0.695 |
| **Physical activity levels (IPAQ)** |  |  |  |
| Low | 2 (12.5%) | 4 (10.0%) | 0.824 |
| Moderate | 9 (56.3%) | 20 (50.0%) |  |
| High | 5 (31.3%) | 16 (40.0%) |  |
| **Rumination (RRS)** | 68.0 (11.9) | 66.1 (11.5) | 0.586 |
| **Self-esteem (RSES)** | 12.8 (5.97) | 11.2 (4.34) | 0.397 |
| **Disability (WHODAS)** | 42.9 (16.1) | 48.1 (12.2) | 0.260 |
| **Motivation (MEI-SF)** | 36.3 (21.9) | 31.2 (10.5) | 0.413 |

*Note.* The table indicates means and standard deviations for age, depressive symptoms, rumination, self-esteem, disability and motivation and for all other variables, total numbers and percentages.

**SM3: Further information exercise therapy**

Logistic mixed model analysis indicated that patients in the CAU+EX condition had a significantly higher likelihood of meeting the exercise prescription, which required engaging in moderate or higher intensity exercise for at least 45 minutes, three times per week, both during the treatment phase (T0-T4), *OR* = 2.05, 95% CI [0.54, 3.57], *p* = .008, and during follow-up (T5-T8), *OR* = 1.23, 95% CI [0.14, 2.33], *p* = .028, compared to CAU alone. Figure 1 displays the number of patients meeting the prescription per condition. We also analysed the impact of the condition on exercise minutes (i.e., sum of the total number of moderate and vigorous intensity leisure time exercise minutes, as reported by the International Physical Activity Questionnaire [IPAQ]). Due to zero-inflation and overdispersion in the data, we employed a zero-inflated negative binomial mixed model. The results revealed that participants in the CAU+EX condition were significantly less likely to report zero exercise minutes during exercise treatment (*OR* = -1.21, 95% CI [-1.66, -0.77], *p* < .001) and during follow-up (*OR* = 1.00, 95% CI [-1.49, -0.51], *p* < .001). However, the effect of the condition on the amount of exercise in the conditional model was not significant during exercise treatment (*b* = 0.34, 95% CI [-0.06, 0.73], *p* = .092) or during follow-up (*b* = 0.08, 95% CI [-0.32, 0.48], *p* = .704).

Additionally, we explored trends in the number of home sessions over time. We found a marginally significant trend indicating a gradual decrease in the number of home sessions over the exercise therapy period (*b* = -0.03, 95% CI [-0.05, 0.00], *p* = .054).

To encourage patients to maintain their exercise routine, we had them assess their mood before and after each session to become aware of the direct mood benefits following exercise. In line with previous research (Basso & Suzuki, 2017), exercise sessions indeed significantly reduced negative mood states (*b* = -9.60, 95% CI [-13.33, -5.88], *p* < .001), and improved positive mood (*b* = 11.49, 95% CI [8.51, 14.47], *p* < .001). The direct mood benefit of an exercise bout did not change over time (*p* = .143 for negative mood and *p* = .237 for positive mood).

Figure 1

*Number of patients meeting the exercise prescription of 135 minutes moderate or vigorous intensity leisure-time exercise per condition during the intervention and follow-up phase*

*Note.* The x-axis represents time of assessment: T0 at baseline, T4 post-exercise-treatment (i.e., 12 weeks), and during follow-up with T5 after 3 months post-treatment, T6 after 6 months post-treatment, T7 after 9 months post-treatment and T8 after 12 months post-treatment. Figure displays raw data.

**SM4: Treatment effectiveness in non-imputed data**

Patients experienced a significant reduction in depressive symptoms over time, *b* = - 1.70, 95% CI [-2.08, -1.32], *p* > .001, however, the time-by-condition interaction was not significant, *b* = -0.02, 95% CI [-0.49, 0.44]*, p* = .916. For depressive remission similarly, the effect of time was significant *OR* = 0.68, 95% CI [0.42, 0.94], *p* > .001, but not the time-by-condition interaction, *OR* = 0.08, 95% CI [-0.26, 0.41]*, p* = .649. Results were similar in the per-protocol analyses, for depressive symptoms the effect of time was significant, *b* = -1.65, 95% CI [-1.99, -1.32], *p* > .001, but not the time-by-condition interaction, *b* = -0.12, 95% CI [-0.58, 0.32], *p* = .593, and for remission similarly, the effect of time was significant, *OR* = 0.75, 95% CI [0.49, 1.00], *p* > .001, but not the time-by-condition interaction, *OR* = -0.05, 95% CI [-0.39, 0.28], *p* = .749.

Figure 2 displays the raw data for each patient in the CAU+EX and CAU condition, which implies individual differences in response to the treatments.

Figure 2

*Depressive symptoms over time for each patient in the CAU+EX and CAU condition*

*Note.* This figure displays the raw scores of depressive symptoms, assessed with the IDS-SR, throughout the study period of 15 months in both conditions separately. T0 refers to baseline, T1 is after 3 weeks of exercise treatment, T2 after 6 weeks, T3 after 9 weeks and T4 after 12 weeks (post-exercise-treatment), T5 is 3 months after T4, T6 is 3 months after T5, T7 is 3 months after T6, and T8 is 3 months after T7.

**SM5: COVID-19 impact on results**

Patients in this study were enrolled from March 2020 to January 2023, which included several periods with social restrictions due to the COVID-19 pandemic. During these periods, psychological treatments were often provided online, fortunately the effectiveness of online and face-to-face treatments are similar (cf. Kambeitz-Ilankovic et al., 2022). Only few patients (n = 3) received individual coaching per telephone (and not face-to-face at the treatment centre) as part of the exercise treatment. We repeated the analyses excluding these participants and the results remained similar. With the imputed data, we found a significant effect of time *b* = -1.02, 95% CI [-1.57, -0.47], *p* = .001 on depressive symptoms, but the time-by-condition interaction was not significant, *b* = -0.18, 95% CI [-0.70, 0.35], *p* = .510. Similarly, for depressive remission, there was a significant effect of time *OR* = 0.73, 95% CI [0.53, 0.93], *p* > .001, but the time-by-condition interaction was not significant, *OR* = 0.08, 95% CI [-0.19, 0.35], *p* = .566. Results were consistent between imputed and non-imputed data.

**SM6: Treatment effectiveness controlled for baseline exercise**

Although both conditions showed no difference in the number of patients meeting the exercise prescription at baseline (so already before inclusion), we reanalyzed our primary outcomes controlling for baseline exercise prescription included as a binary predictor. This was to ensure that any non-significant baseline differences in exercise did not affect the results. The findings remained consistent: with the imputed data, we observed a significant reduction in depressive symptoms over time (*b* = -1.02, 95% CI [-1.56, -0.47], *p* = .001), with no significant interaction between time and condition for depressive symptoms (*b* = -0.22, 95% CI [-0.72, 0.28], *p* = .393), and a similar pattern for remission (time: *OR* = 0.82, 95% CI [0.64, 1.00], *p* > .001 and time-by-condition: *b* = -0.11, 95% CI [-0.26, 0.04] , *p* = .149). Results were consistent between imputed and non-imputed data.

**SM7: Treatment effectiveness on IDS-SR subscales**

The IDS-SR measures diverse aspects of depressive symptom severity. Therefore, we also analysed the effects of treatment condition on its three subscales, which were previously identified through confirmatory factor analyses (Wardenaar et al., 2010). These subscales assess mood/cognition, anxiety/arousal, and sleep (Wardenaar et al., 2010). We conducted separate analyses for each subscale using the same linear mixed-effects models as for the total IDS-SR score (see manuscript for detailed methods). While all subscales showed significant reductions in depressive symptoms over time, none of the time-by-condition interactions were significant (all *p* > .05). Results were consistent between imputed and non-imputed data.

**SM8: The number of exercise sessions as predictor of depressive symptoms**

To explore whether a higher number of exercise sessions (i.e., the sum of supervised and home-based sessions) predicts greater reductions in depressive symptoms over time within the CAU+EX condition, we conducted a mixed-model analysis. Depressive symptoms were the outcome variable, with time, the cumulative number of weekly exercise sessions (i.e., the total sessions after 3, 6, 9, and 12 weeks; T1-T4 with baseline values set to 0 at T0), and their interaction included as predictors, with a random effect for patients nested within treatment centers. The analysis revealed no significant interaction between exercise sessions and time, b = 0.59, 95% CI [-0.12, 1.28], p = .102. Consequently, these findings do not directly inform dose recommendations for exercise therapy in specialized mental health care.

**SM9: Exploratory moderator**

We conducted separate linear mixed-effects model analyses to examine whether age, sex, or educational level moderated the effect of treatment condition on depressive symptoms. The models included depressive symptoms as the outcome, with fixed effects for time, the moderator variable (age, sex, or educational level), treatment condition, and their interactions, with a random effect for patients nested within treatment centers. None of the variables—age, sex, or educational level—emerged as significant moderators of the treatment effect (all *p* > .05).

Similarly, we explored whether any baseline variables might be associated with treatment response (defined as a ≥50% reduction on the IDS-SR) within the adjunct exercise condition. We conducted separate logistic regression analyses for each baseline variable (i.e., age, sex, presence of a somatic disorder, comorbid psychological disorder, meeting the exercise prescription of ≥135 min/week of moderate- to vigorous-intensity leisure-time exercise, depressive symptoms, disability, or rumination), with response (coded as 0 or 1) as the dependent variable, using the multiply imputed datasets. No baseline variables were significantly associated with response. However, the exploratory results suggested possible trends indicating that females and patients with higher baseline depressive symptoms, disability, or somatic disorders might be less likely to benefit, whereas patients with a comorbid psychological diagnosis might be more likely to respond (see Table 3). The results were similar when the analyses were conducted on the raw (non-imputed) dataset. Given the limited power of this analysis, these preliminary findings should be interpreted with caution but may help to generate hypotheses for future research examining who is more or less likely to benefit from adjunct exercise therapy.

Table 3

*Exploratory logistic regression results for baseline predictors of treatment response (≥50% IDS-SR reduction) in the adjunct exercise condition (multiply imputed datasets)*

| **Predictor** | **Responders**  **N = 16** | | **Non-Responders**  **N = 40** | **Estimate**  **(95% CI)** | ***p*** |
| --- | --- | --- | --- | --- | --- |
| Age in years, Mean (SD) | | 35.50 (12.58) | 37.43 (13.43) | 0.99 (0.94–1.04) | .641 |
| Sex (% Female) | | 44% | 52% | 1.37 (0.41–4.55) | .609 |
| Somatic Disorder (% Yes) | | 25% | 30% | 0.76 (0.17–3.43) | .715 |
| Comorbid diagnoses (% Yes) | | 61% | 43% | 2.09 (0.46–9.50) | .328 |
| Exercise prescription (% Yes) | | 21% | 17% | 1.33 (0.28–6.35) | .717 |
| Depressive symptoms Mean (SD) | | 40.29 (10.56) | 43.09 (11.16) | 0.98 (0.91–1.05) | .492 |
| Disability Mean (SD) | | 42.26 (12.13) | 48.35 (13.74) | 0.96 (0.91–1.02) | .220 |
| Rumination Mean (SD) | | 66.25 (7.98) | 66.77 (12.80) | 1.00 (0.94–1.05) | .884 |

*Note.* Results are pooled across multiple imputations. The number of responders varied across the five imputed data sets; here, the mean N is presented. The reference group for sex is male. Approximately 29.1% of participants were classified as responders in the adjusted exercise condition. Comorbid diagnoses refer to the presence of comorbid psychological diagnoses.

**SM10: Treatment effectiveness on secondary outcomes**

Given the potential broader impact of exercise treatment, we included several secondary outcomes and assessed treatment effectiveness using mixed-effects models. The condition-by-time interaction was the predictor, as well as the main effect of time (Twisk et al., 2018), with a random effect for patients clustered within treatment centres. We assessed the following secondary outcomes: general physical activity using the Dutch version of the International Physical Activity Questionnaire (IPAQ; Vandelanotte et al., 2005), disability with the Dutch version of the World Health Organization Disability Assessment Schedule (WHODAS 2.0; Ustun et al., 2010), motivation and energy with the Motivation and Energy Inventory-Short Form (MEI-SF; Fehne et al., 2004), rumination with the Dutch version of the Ruminative Response Scale (RRS; Raes et al., 2003), self-esteem with the Dutch version of the Rosenberg self-esteem scale (RSES; Franck et al., 2008), and explicit memory bias with the computerized Self-Referent Encoding Task (SRET; Derry & Kuiper, 1981). For a comprehensive description of outcomes and the assessment schedule, we refer to the published protocol paper (Schmitter et al., 2020). Table 4 presents the results for both the imputed and non-imputed data, including analyses for the full sample and the per-protocol sample.

General physical activity levels followed a non-linear pattern over time, so time was coded using dummy variables (Twisk et al., 2018). As the assumptions for a linear mixed model were not met, we also fitted a zero-inflated model with a negative binomial family and log link. Since the results were similar, we report the linear model to align with our protocol. None of the dummy variables for time or the time-by-condition interactions were significant (all *p* > .05), indicating no significant improvement in physical activity over time, regardless of condition. Similarly, for the SRET which indicates the ratio of self-relevant negative words recalled to all self-relevant recalled words (i.e., explicit negative memory bias), we fitted a quasibinomial model which is better suited for these outcomes (Gómez–Déniz et al., 2020). Yet, results remained similar and therefore we report the results of the linear models (see Table 3).

Additionally, physical fitness was objectively assessed using a submaximal VO2_max_ test, conducted by a blinded assessor at both baseline and post-exercise-treatment (after 12 weeks). Initially, the goal was to perform the test on 15% of the sample (n = 18), with the primary endpoint being an improvement in cardiorespiratory fitness. Due to COVID-19 restrictions, baseline testing was completed by only 13 participants in the CAU+EX condition and 7 in the CAU condition, with only 4 participants in the CAU condition attending the post-treatment assessment. To evaluate the treatment's effect on physical fitness, a mixed model analysis was conducted with time and the time-by-condition interaction as predictors, without imputing missing values due to the small sample size. The analysis revealed no significant effect of time or a time-by-condition interaction (all *p* > .05, CAU+EX: *M_pre_* = 35.5 and *M_post_ =* 36.7; CAU: *M_pre_* = 27.8 and *M_post_ =* 33.6). However, these findings should be interpreted with caution, as the analysis was likely underpowered.

Table 4

|  |  | **Imputed** | | | **Non-imputed** | | | **Per-protocol imputed** | | | | | **Per-protocol non- imputed** | | |
| --- | --- | --- | --- | --- | --- | --- | --- | --- | --- | --- | --- | --- | --- | --- | --- |
| **Outcome** | **Effect** | ***b*** | ***p*** | ***95% CI*** | ***b*** | ***p*** | ***95% CI*** | | ***b*** | ***p*** | ***95% CI*** | ***b*** | | ***p*** | ***95% CI*** |
| Disability | Time | -0.69 | .063 | [-1.43, 0.04] | -1.21 | >.001 | [-1.95,-0.50] | | -0.57 | .355 | [-1.93, 0.79] | -1.17 | | >.001 | [-1.82, -0.52] |
|  | Time x condition | -0.50 | .341 | [-1.56, 0.56] | -0.64 | .155 | [-1.52, 0.24] | | -0.90 | .073 | [-1.89, 0.09] | -0.86 | | .051 | [-1.74, -0.01] |
| Motivation | Time | 1.11 | .007 | [0.31, 1.89] | 1.68 | >.001 | [0.96, 2.40] | | 1.07 | .040 | [0.06, 2.08] | 1.76 | | >.001 | [1.09, 2.44] |
|  | Time x condition | 0.26 | .663 | [-0.98, 1.49] | 0.27 | .549 | [-0.62, 1.13] | | 0.44 | .265 | [-0.33, 1.21] | 0.16 | | .723 | [-0.71, 1.03] |
| Rumination | Time | -1.10 | .001 | [-1.70, -0.49] | -1.66 | >.001 | [-2.08, -.1.25] | | -1.01 | >.001 | [-1.50, -0.52] | -1.64 | | >.001 | [-2.01, -1.27] |
|  | Time x condition | -0.32 | .365 | [-1.10, 0.47] | -0.16 | .533 | [-0.67, 0.35] | | -0.50 | .113 | [-1.12, 0.13] | -0.23 | | .353 | [-0.73, 0.26] |
| Self-esteem | Time | 0.19 | .254 | [-0.17, 0.54] | 0.42 | >.001 | [0.26, 0.58] | | 0.21 | .052 | [-0.00, 0.43] | 0.46 | | >.001 | [0.31, 0.61] |
|  | Time x condition | 0.02 | .827 | [-0.19, 0.23] | 0.04 | .716 | [-0.16, 0.24] | | 0.06 | .642 | [-0.22, 0.34] | 0.03 | | .798 | [-0.17, 0.22] |
| Memory bias | Time | -0.01 | .590 | [-0.02, 0.01] | -0.03 | .055 | [-0.06, 0.00] | | -0.01 | .161 | [-0.02, 0.00] | -0.00 | | .660 | [-0.05, 0.00] |
|  | Time x condition | 0.00 | .874 | [-0.02, 0.02] | 0.01 | .414 | [-0.02, 0.05] | | 0.00 | .559 | [-0.01, 0.01] | -0.00 | | .886 | [-0.03, 0.04] |

*Treatment effectiveness on secondary outcomes*

*Note.* Disability was assessed with the Dutch version of the World Health Organization Disability Assessment Schedule (WHODAS 2.0; Ustun et al., 2010), motivation and energy with the Motivation and Energy Inventory-Short Form (MEI-SF; Fehne et al., 2004), rumination with the Dutch version of the Ruminative Response Scale (RRS; Raes et al., 2003), self-esteem with the Dutch version of the Rosenberg self-esteem scale (RSES; Franck et al., 2008), and explicit memory bias with the computerized Self-Referent Encoding Task (SRET; Derry & Kuiper, 1981). The SRET had many missing values (CAU T0: 52.63%, T4: 78.95%, T8: 81.58%; CAU+EX T0: 44.64%, T4: 60.71%, T8: 85.71%) due to frequent program download failures on home computers. Results should be interpreted with caution.

**SM11: Sensitivity analyses economic evaluation**

As sensitivity analyses, we repeated the economic evaluation with treatment response

instead of QALY as outcome. The reduction in IDS‐SR scores was dichotomized in either less or more than 50% to calculate the incremental costs per responder. Moreover, we assessed the cost-effectiveness from a healthcare perspective instead of a societal perspective (excluding patient and family costs and productivity losses), and with winsorized costs where values above the 95th percentile were replaced with the 95th percentile value, to deal with possible outliers. Analyses were the same as on our primary outcomes reported in the main paper. The incremental cost-utility ratio (ICUR) was determined using the differences in costs and QALYs between CAU and CAU+EX. Bootstrapped ICERs/ICURs were plotted on a cost-effectiveness plane, and a cost-effectiveness acceptability curve (CEAC) was generated to assess the likelihood of the exercise treatment being cost-effective at various willingness-to-pay (WTP) values per QALY. For this study, a WTP threshold of €50,000 per QALY was assumed for moderate to severe depression (Vijgen, 2018).

***Treatment response***

The cost-effectiveness analysis based on bootstrapped data showed a response difference of 0.009, CI [-0.25, 0.26] in favour of CAU+EX, with CAU+EX incurring an additional cost of €4,054 CI [-1998.43, 10093.29]. This resulted in ICUR of €453,179.90. The probability that the ICER was acceptable cannot be stated, because the WTP threshold is unknown. Given that the difference in clinical response between CAU+EX and CAU alone is minimal, and the additional costs associated with CAU+EX are relatively high, the cost-effectiveness of adding exercise treatment to CAU is questionable. The cost-effectiveness plane and CEAC are shown in Figure 3.

Figure 3

*The cost-effectiveness plane (A) and cost-utility acceptability curve (B) for response rate*

*
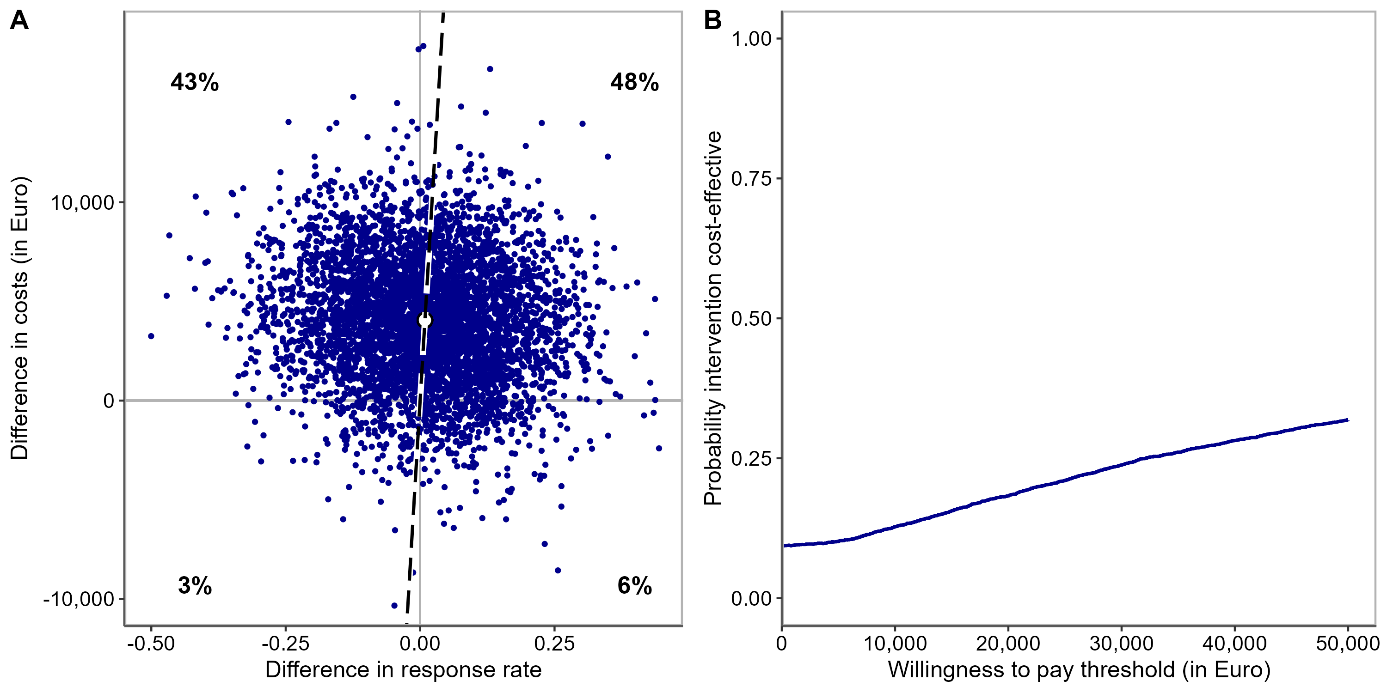
*

*Note.* The cost-effectiveness plane (A) illustrates the incremental costs and effects of CAU+EX compared to CAU. The dashed line represents the mean incremental cost-effectiveness ratio (ICER) for the comparison. The cost-utility acceptability curve (B) shows the probability that the exercise treatment is cost-effective across a range of WTP thresholds, up to €50,000 per additional QALY gain.

***Healthcare perspective***

The cost-utility analysis from a healthcare perspective based on bootstrapped data showed a difference in QALY of 0.004, CI [-0.09, 0.11] in favour of CAU+EX, with CAU+EX incurring an additional cost of €437.54 CI [-3030.17, 3968.81]. This resulted in ICUR of €109,953.40, which exceeds the WTP threshold of €50,000. The cost-effectiveness plane and CEAC are shown in Figure 4. The CEAC indicated that the probability of CAU+EX being cost-effective at a WTP threshold of €50,000 was 48%.

Figure 4

*The cost-effectiveness plane (A) and cost-utility acceptability curve (B) from a healthcare perspective*

*
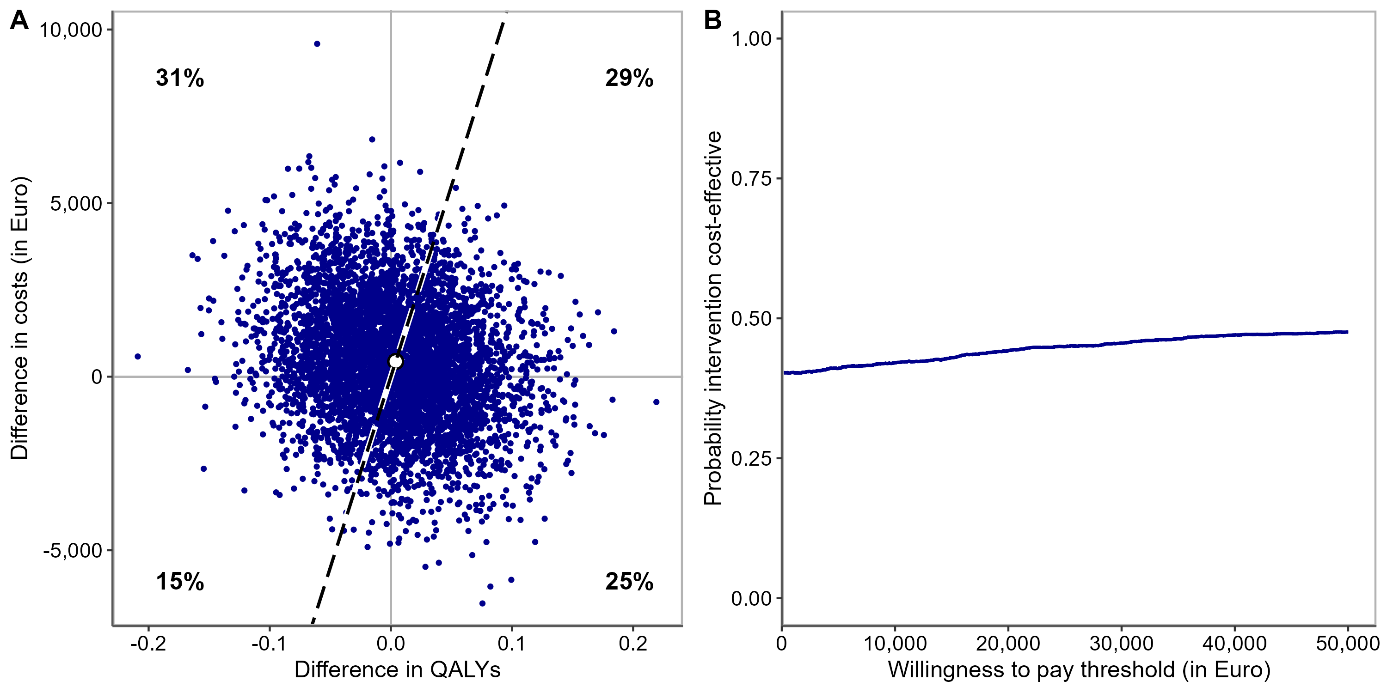
*

*Note.* The cost-effectiveness plane (A) illustrates the incremental costs and effects of CAU+EX compared to CAU. The dashed line represents the mean incremental cost-effectiveness ratio (ICER) for the comparison. The cost-utility acceptability curve (B) shows the probability that the exercise treatment is cost-effective across a range of WTP thresholds, up to €50,000 per additional QALY gain.

***Winsorized costs***

The cost-utility analysis with costs being winsorized based on bootstrapped data showed a difference in QALY of 0.004, CI [-0.09, 0.11] in favour of CAU+EX, with CAU+EX incurring an additional cost of €3014.38 CI [-2471.82, 8801.83]. This resulted in ICUR of €757,512.60, which exceeds the WTP threshold of €50,000. The cost-effectiveness plane and CEAC are shown in Figure 5. The CEAC indicated that the probability of CAU+EX being cost-effective at a WTP threshold of €50,000 was 25%.

Figure 5

*The cost-effectiveness plane (A) and cost-utility acceptability curve (B) with winsorized costs*

***
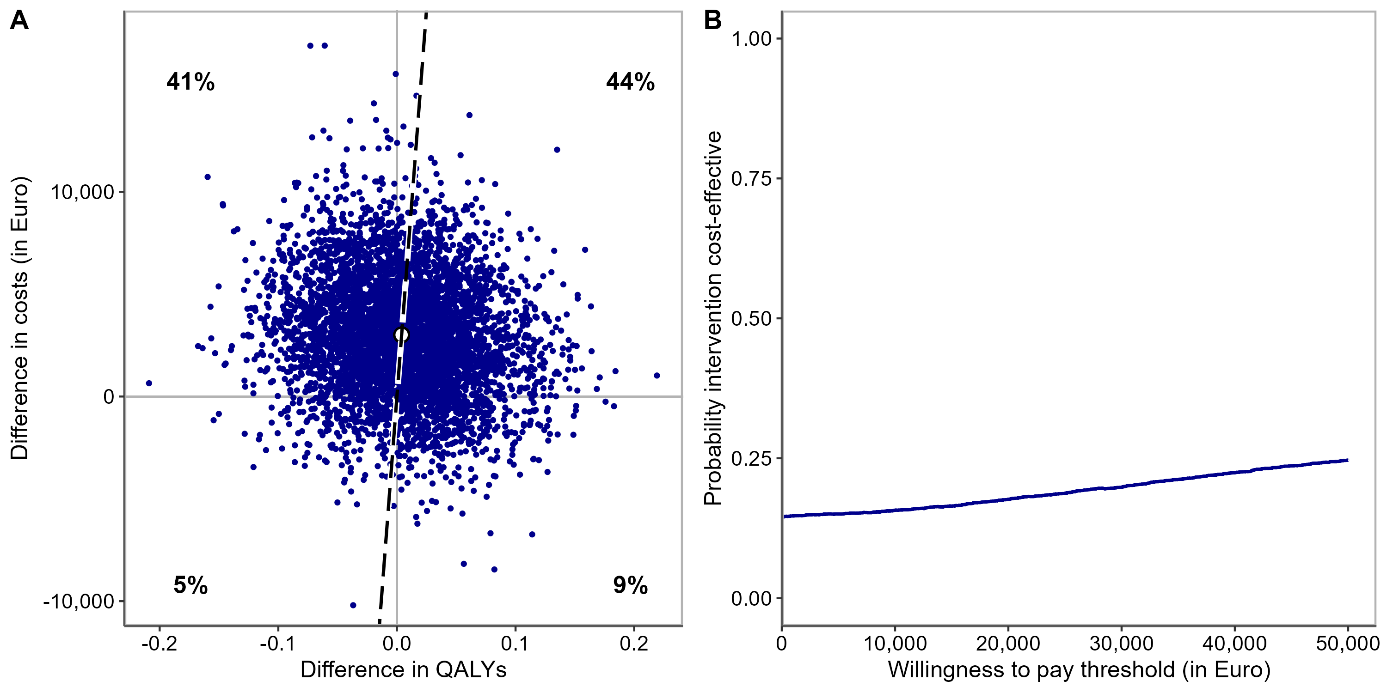
***

*Note.* The cost-effectiveness plane (A) illustrates the incremental costs and effects of CAU+EX compared to CAU. The dashed line represents the mean incremental cost-effectiveness ratio (ICER) for the comparison. The cost-utility acceptability curve (B) shows the probability that the exercise treatment is cost-effective across a range of WTP thresholds, up to €50,000 per additional QALY gain.

**SM12: Cost-effectiveness plane and CEAC in per-protocol sample**

Figure 5

*The cost-effectiveness plane (A) and cost-utility acceptability curve (B) in per-protocol sample*

*
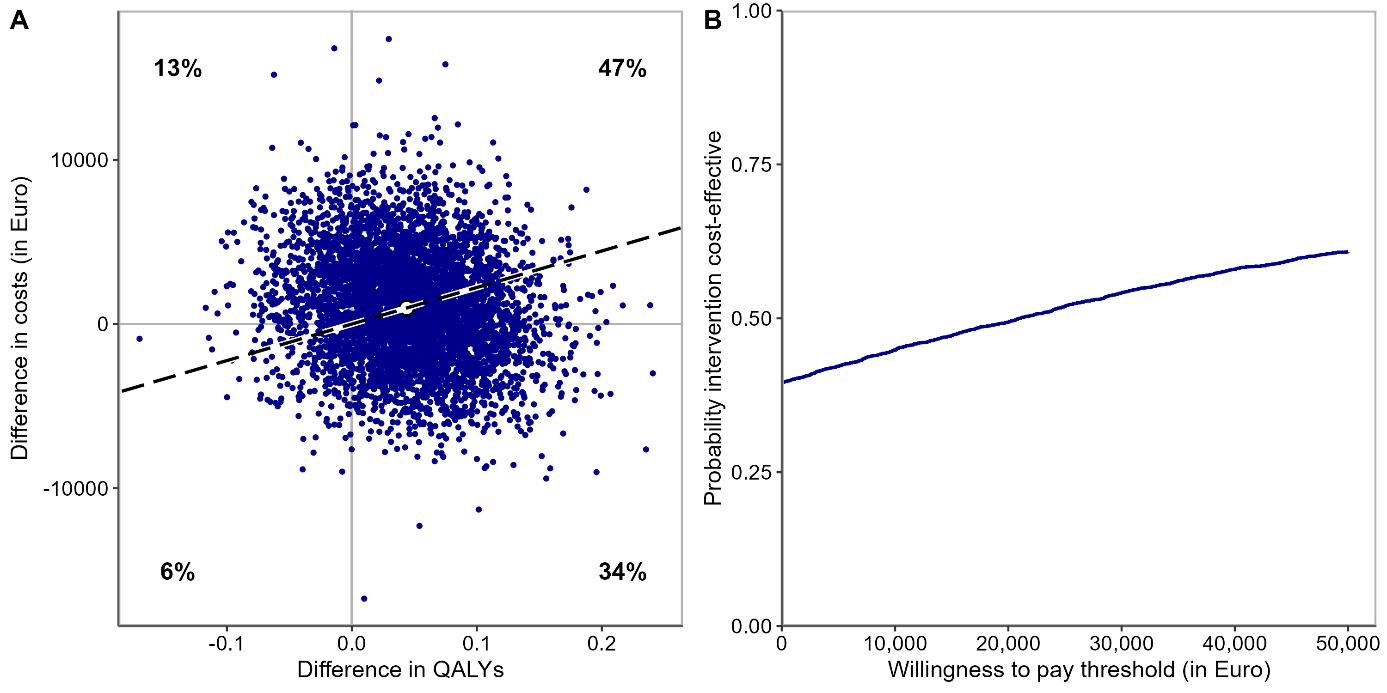
*

*Note.* The cost-effectiveness plane (A) illustrates the incremental costs and effects of CAU+EX compared to CAU. The dashed line represents the mean incremental cost-effectiveness ratio (ICER) for the comparison. The cost-utility acceptability curve (B) shows the probability that the exercise treatment is cost-effective across a range of WTP thresholds, up to €50,000 per additional QALY gain.

**SM13. Additional information cost-effectiveness analysis**

Table 5

*Mean costs (in Euros) and 95% confidence intervals of CAU+EX and CAU at baseline and 3, 6, 9, 12 and 15 months follow-up over a four-week period, as well as bootstrapped cumulative cost*

|  | | **Baseline^a^** | | | | | **3 Months^a^** | | | | | **6 Months^a^** | | | | **9 Months^a^** | | | | **12 Months^a^** | | | | | **15 Months^a^** | | | | |
| --- | --- | --- | --- | --- | --- | --- | --- | --- | --- | --- | --- | --- | --- | --- | --- | --- | --- | --- | --- | --- | --- | --- | --- | --- | --- | --- | --- | --- | --- |
|  | **EX+CAU (N=56)** | | **CAU**  **(N=38)** | | **EX+CAU (N=44)** | | | **CAU (N=25)** | | **EX+CAU (N=38)** | | | **CAU (N=20)** | | **EX+CAU (N=36)** | | **CAU (N=22)** | | **EX+CAU (N=34)** | | | **CAU (N=21)** | | **EX+CAU (N=32)** | | | **CAU**  **(N=21)** |  |  |
| **Healthcare utilization** | | | |  | |  | | |  | |  | | |  | |  | |  | | |  | |  | | |  | |  |  |
| Primary Care^b^ | 224 ± 384 | | 297 ± 607 | | 178 ± 555 | | | 128 ± 193 | | 223 ± 566 | | | 83.7 ± 120 | | 134 ± 351 | | 191 ± 269 | | 113 ± 290 | | | 136 ± 238 | | 267 ± 728 | | | 87.1 ± 185 | |  |
| Mental Care^c^ | 649 ± 701 | | 605 ± 536 | | 717 ± 522 | | | 817 ± 584 | | 537 ± 481 | | | 344 ± 273 | | 405 ± 374 | | 435 ± 355 | | 347 ± 319 | | | 397 ± 411 | | 420 ± 466 | | | 473 ± 634 | |  |
| Other Care^d^ | 159 ± 238 | | 127 ± 164 | | 129 ± 162 | | | 90.0 ± 79.1 | | 97.8 ± 103 | | | 102 ± 107 | | 78.2 ± 92.3 | | 87.3 ± 128 | | 81.3 ± 112 | | | 86.2 ± 128 | | 120 ± 151 | | | 90.5 ± 107 | |  |
| Help at Home^e^ | 28.0 ± 122 | | 22.4 ± 138 | | 47.8 ± 161 | | | 45.0 ± 172 | | 30.2 ± 106 | | | 6.84 ± 21.1 | | 26.2 ± 99.2 | | 112 ± 525 | | 17.1 ± 59.7 | | | 11.4 ± 35.2 | | 9.77 ± 44.8 | | | 15.0 ± 53.5 | |  |
| Medication^f^ | 14.2 ± 9.59 | | 12.9 ± 10.9 | | 13.8 ± 8.33 | | | 13.0 ± 10.7 | | 14.9 ± 9.45 | | | 13.7 ± 7.75 | | 11.7 ± 9.24 | | 13.9 ± 10.8 | | 10.5 ± 9.26 | | | 12.9 ± 10.9 | | 13.7 ± 9.59 | | | 9.66 ± 9.06 | |  |
| Total  (95% CI) | 1050  (101–3982) | | 1040  (23–3345) | | 1040  (263–3196) | | | 1050  (32–2481) | | 872  (101–3982) | | | 543  (141–2407) | | 629  (6–1131) | | 727  (22–2078) | | 552  (0–1768) | | | 632  (100–1661) | | 821  (34–2741) | | | 660  (6–2777) |  |  |
| **Informal Care** | | |  | |  | | |  | |  | | |  | |  | |  | |  | | |  | |  | | |  |  |  |
| Travel | 33.7 ± 32.0 | | 28.8 ± 24.7 | | 32.0 ± 22.8 | | | 31.8 ± 20.3 | | 25.3 ± 17.9 | | | 15.7 ± 11.5 | | 18.7 ± 15.4 | | 20.1 ± 17.1 | | 16.9 ± 15.6 | | | 19.6 ± 18.1 | | 19.1 ± 17.1 | | | 23.7 ± 24.0 | |  |
| Family Friends | 151 ± 307 | | 236 ± 524 | | 129 ± 447 | | | 95.8 ± 165 | | 171 ± 478 | | | 82.1 ± 150 | | 103 ± 300 | | 140 ± 212 | | 80.5 ± 249 | | | 83.6 ± 209 | | 208 ± 598 | | | 55.6 ± 141 | |  |
| Total  (95% CI) | 185  (4–1171) | | 265  (4–2063) | | 161  (5–1101) | | | 128  (4–585) | | 196  (4–984) | | | 97.8  (0–458) | | 121  (0–703) | | 160  (7–597) | | 97.3  (0–559) | | | 103  (6–662) | | 228  (2–502) | | | 79.2  (0–1626) |  |  |
| **Productivity losses** | | | |  | |  | | |  | |  | | |  | |  | |  | | |  | |  | | |  | |  |  |
| Paid Work, Absenteeism | 532 ± 1410 | | 996 ± 2120 | | 351 ± 1450 | | | 174 ± 815 | | 582 ± 2350 | | | 11.9 ± 53.2 | | 130 ± 315 | | 0 ± 0 | | 54.9 ± 239 | | | 71.7 ± 245 | | 96.5 ± 260 | | | 232 ± 1140 | |  |
| Paid Work, Presenteeism | 731 ± 1430 | | 574 ± 1310 | | 421 ± 1050 | | | 155 ± 377 | | 390 ± 884 | | | 65.5 ± 152 | | 149 ± 358 | | 97.9 ± 189 | | 103 ± 275 | | | 108 ± 250 | | 70.4 ± 120 | | | 224 ± 529 | |  |
| Unpaid Work, Absenteeism | 0 ± 0 | | 1.13 ± 6.93 | | 0 ± 0 | | | 0 ± 0 | | 0 ± 0 | | | 0 ± 0 | | 3.80 ± 17.9 | | 1.94 ± 6.43 | | 1.81 ± 10.6 | | | 1.90 ± 8.06 | | 7.87 ± 25.6 | | | 4.81 ± 19.0 | |  |
| Unpaid Work, Presenteeism | 3.97 ± 20.4 | | 1.35 ± 4.67 | | 2.72 ± 11.6 | | | 18.5 ± 69.1 | | 5.40 ± 22.5 | | | 125 ± 535 | | 80.8 ± 429 | | 6.22 ± 22.7 | | 3.52 ± 7.02 | | | 28.0 ± 80.4 | | 32.6 ± 131 | | | 4.81 ± 8.94 | |  |
| Total  (95% CI) | 1270  (0–8414) | | 1570  (0–9644) | | 774  (0–4371) | | | 348  (0–2458) | | 978  (0–7359) | | | 202  (0–1558) | | 364  (0–2549) | | 106  (0–570) | | 163  (0–1313) | | | 210  (0–1348) | | 207  (0–3878) | | | 466  (0–949) |  |  |
| **Societal costs** | | |  | |  | | |  | |  | | |  | |  | |  | |  | | |  | |  | | |  |  |  |
| Total  (95% CI) | 2530  (142–9787) | | 2900  (193–12347) | | 2020  (271–7728) | | | 1570 (273–4509) | | 2080  (191–7838) | | | 850  (49–2371) | | 1140  (52–3534) | | 1100  (197–3407) | | 830  (17–2659) | | | 956  (199–2299) | | 1270  (50–4834) | | | 1220  (29–4836) |  |  |

*Note*. CI, Confidence Interval. Values represent mean ± SD, unless otherwise indicated. The Table displays raw (non-imputed) data.

^a^ Health care utilization, informal care and productivity costs of the past 4 weeks.

^b^ Contact with the general practitioner, or practice nurse.

^c^ Contact with psychiatrist, psychiatric nurse, psychologist, or social worker.

^d^ Contact with physiotherapist, dietician, or alternative healer.

^e^ Home care of family assistance, or other paid help.

^f^ Antidepressants, anti-anxiety, or sleeping pills.

**SM 14. Consort and Cheers checklists**

Both checklist display the page numbers of the submitted manuscript.

Figure 6

*CONSORT 2010 checklist of information to include when reporting a randomised trial**

| Section/Topic | Item No | Checklist item | Reported on page No | |
| --- | --- | --- | --- | --- |
| Title and abstract | | | | |
|  | 1a | Identification as a randomised trial in the title | | 1 |
|  | 1b | Structured summary of trial design, methods, results, and conclusions (for specific guidance see CONSORT for abstracts) | | 2-3 |
| Introduction | | | | |
| Background and objectives | 2a | Scientific background and explanation of rationale | | 4-5 |
|  | 2b | Specific objectives or hypotheses | | 5 |
| Methods | | | | |
| Trial design | 3a | Description of trial design (such as parallel, factorial) including allocation ratio | | 5 |
|  | 3b | Important changes to methods after trial commencement (such as eligibility criteria), with reasons | | 6 |
| Participants | 4a | Eligibility criteria for participants | | 6 |
|  | 4b | Settings and locations where the data were collected | | 5 |
| Interventions | 5 | The interventions for each group with sufficient details to allow replication, including how and when they were actually administered | | 7-8 |
| Outcomes | 6a | Completely defined pre-specified primary and secondary outcome measures, including how and when they were assessed | | 8-10 |
|  | 6b | Any changes to trial outcomes after the trial commenced, with reasons | | protocol paper: doi: 10.1186/s12888-020-02989-z |
| Sample size | 7a | How sample size was determined | | 6 |
|  | 7b | When applicable, explanation of any interim analyses and stopping guidelines | | 6 |
| Randomisation: |  |  | |  |
| Sequence generation | 8a | Method used to generate the random allocation sequence | | 6-7 |
|  | 8b | Type of randomisation; details of any restriction (such as blocking and block size) | | 6-7 |
| Allocation concealment mechanism | 9 | Mechanism used to implement the random allocation sequence (such as sequentially numbered containers), describing any steps taken to conceal the sequence until interventions were assigned | | 6-7 |
| Implementation | 10 | Who generated the random allocation sequence, who enrolled participants, and who assigned participants to interventions | | 6-7 |
| Blinding | 11a | If done, who was blinded after assignment to interventions (for example, participants, care providers, those assessing outcomes) and how | | 7 |
|  | 11b | If relevant, description of the similarity of interventions | | NA |
| Statistical methods | 12a | Statistical methods used to compare groups for primary and secondary outcomes | | 10-11 |
|  | 12b | Methods for additional analyses, such as subgroup analyses and adjusted analyses | | SM |
| Results | | | | |
| Participant flow (a diagram is strongly recommended) | 13a | For each group, the numbers of participants who were randomly assigned, received intended treatment, and were analysed for the primary outcome | | 12 |
|  | 13b | For each group, losses and exclusions after randomisation, together with reasons | | 11-12 |
| Recruitment | 14a | Dates defining the periods of recruitment and follow-up | | 7 |
|  | 14b | Why the trial ended or was stopped | | NA |
| Baseline data | 15 | A table showing baseline demographic and clinical characteristics for each group | | 12 |
| Numbers analysed | 16 | For each group, number of participants (denominator) included in each analysis and whether the analysis was by original assigned groups | | 12 |
| Outcomes and estimation | 17a | For each primary and secondary outcome, results for each group, and the estimated effect size and its precision (such as 95% confidence interval) | | 12-14 |
|  | 17b | For binary outcomes, presentation of both absolute and relative effect sizes is recommended | | 12-14 |
| Ancillary analyses | 18 | Results of any other analyses performed, including subgroup analyses and adjusted analyses, distinguishing pre-specified from exploratory | | 12-14 |
| Harms | 19 | All important harms or unintended effects in each group (for specific guidance see CONSORT for harms) | | 14 |
| Discussion | | | | |
| Limitations | 20 | Trial limitations, addressing sources of potential bias, imprecision, and, if relevant, multiplicity of analyses | | 15-18 |
| Generalisability | 21 | Generalisability (external validity, applicability) of the trial findings | | 15-18 |
| Interpretation | 22 | Interpretation consistent with results, balancing benefits and harms, and considering other relevant evidence | | 15-18 |
| Other information | | | |  |
| Registration | 23 | Registration number and name of trial registry | | 5 |
| Protocol | 24 | Where the full trial protocol can be accessed, if available | | 5 |
| Funding | 25 | Sources of funding and other support (such as supply of drugs), role of funders | | 20 |

*We strongly recommend reading this statement in conjunction with the CONSORT 2010 Explanation and Elaboration for important clarifications on all the items. If relevant, we also recommend reading CONSORT extensions for cluster randomised trials, non-inferiority and equivalence trials, non-pharmacological treatments, herbal interventions, and pragmatic trials. Additional extensions are forthcoming: for those and for up to date references relevant to this checklist, see [www.consort-statement.org](http://www.consort-statement.org). NA not applicable, SM supplementary material.

Figure 7

**
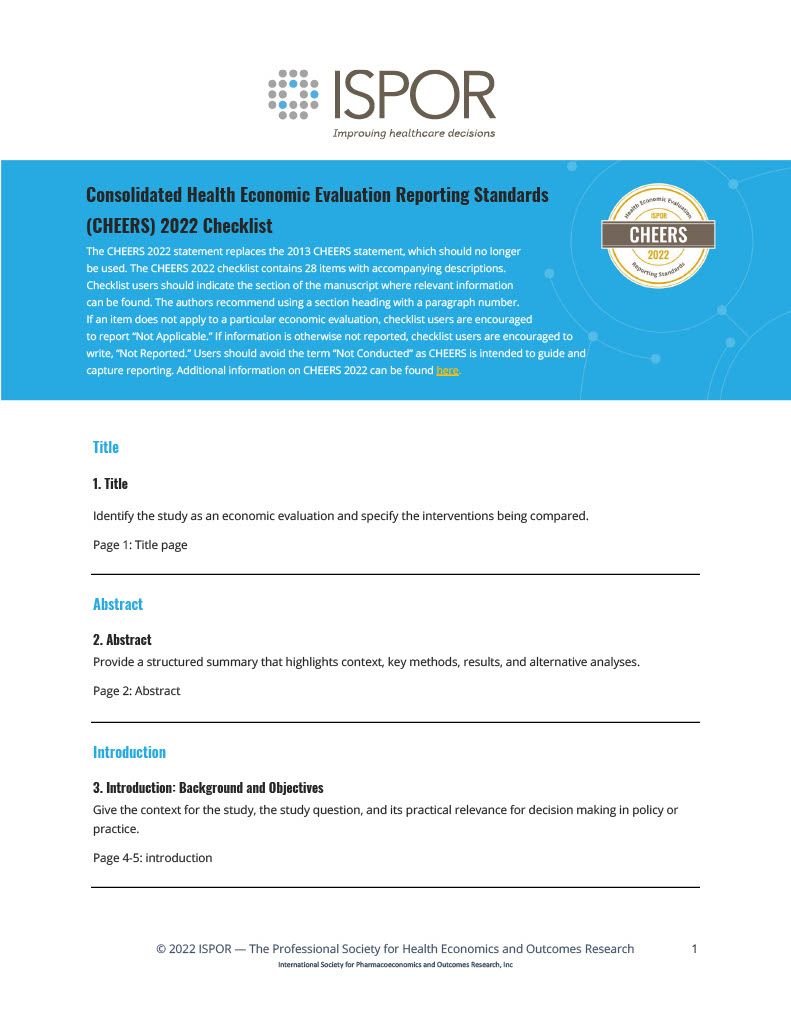
***Consolidated Health Economic Evaluation Reporting Standards (CHEERS) 2022 Checklist*

**
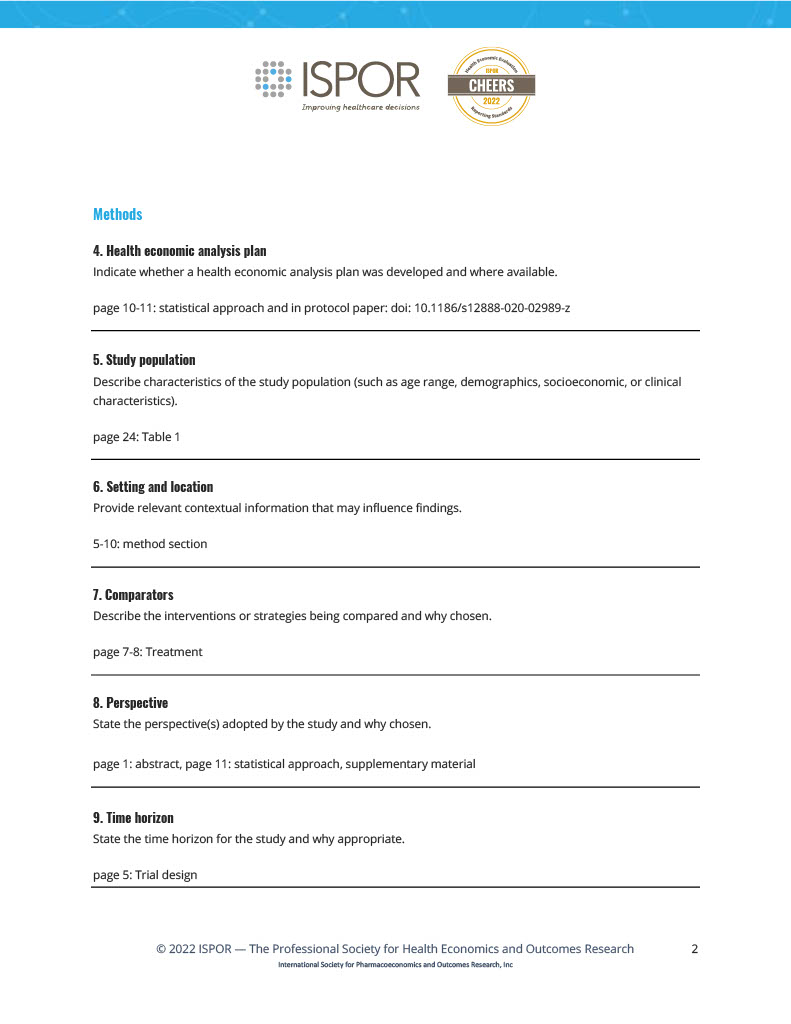
**

**
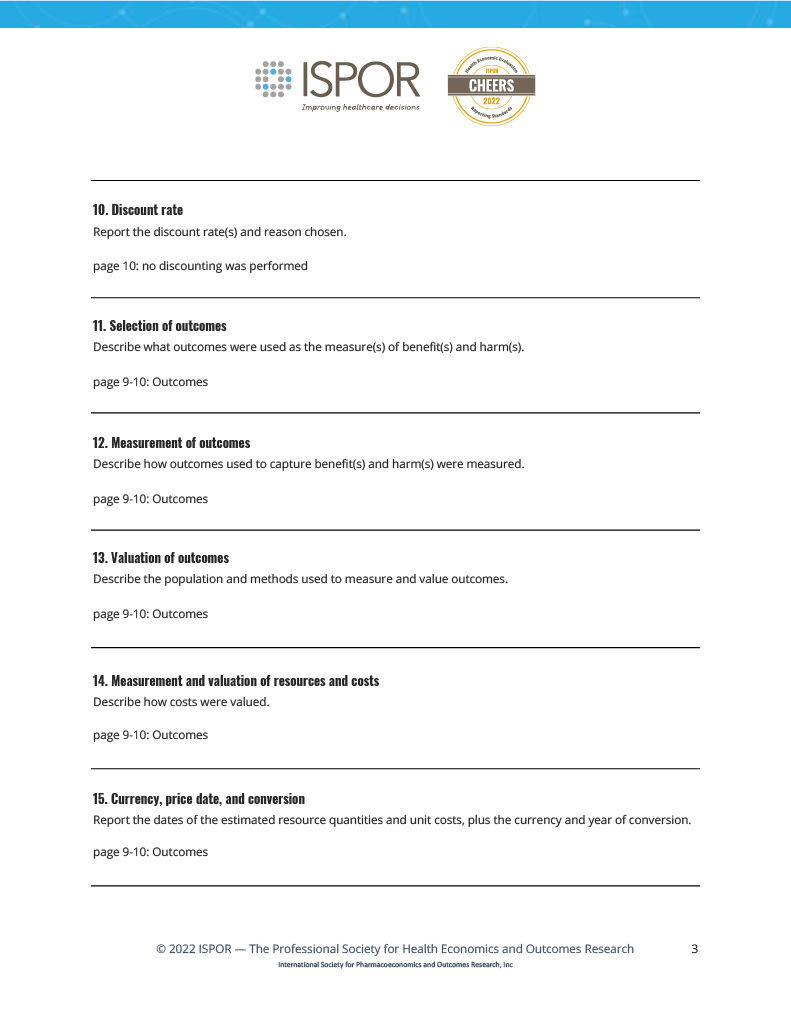
**

**
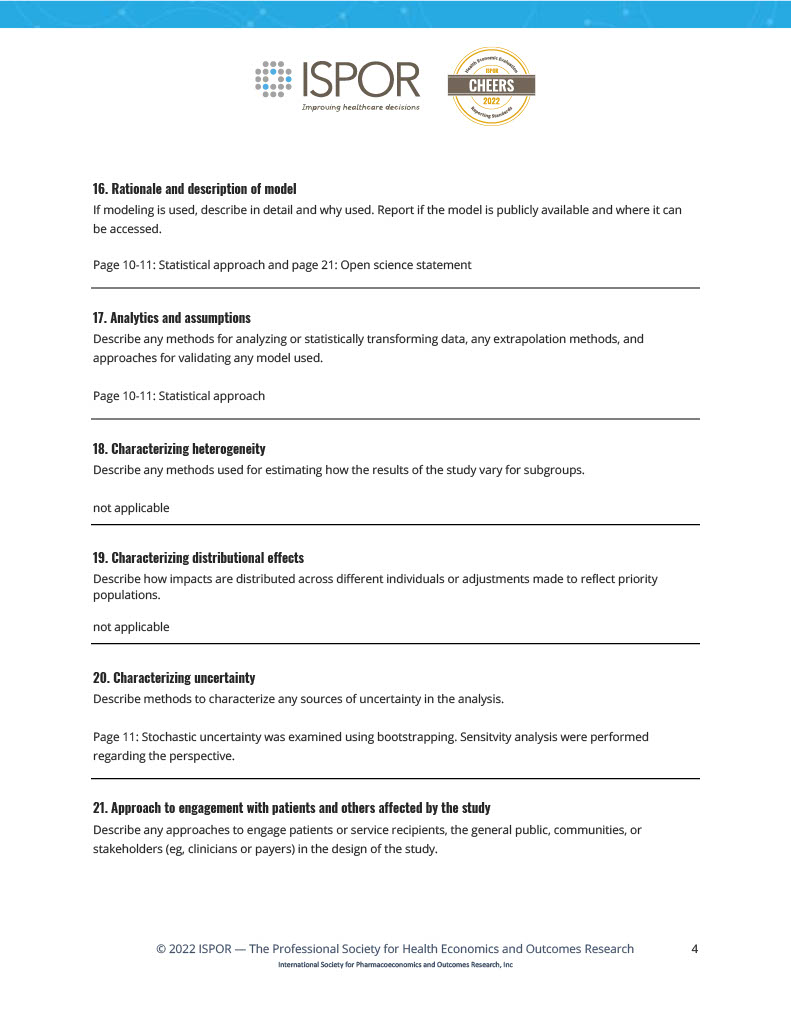
**

**
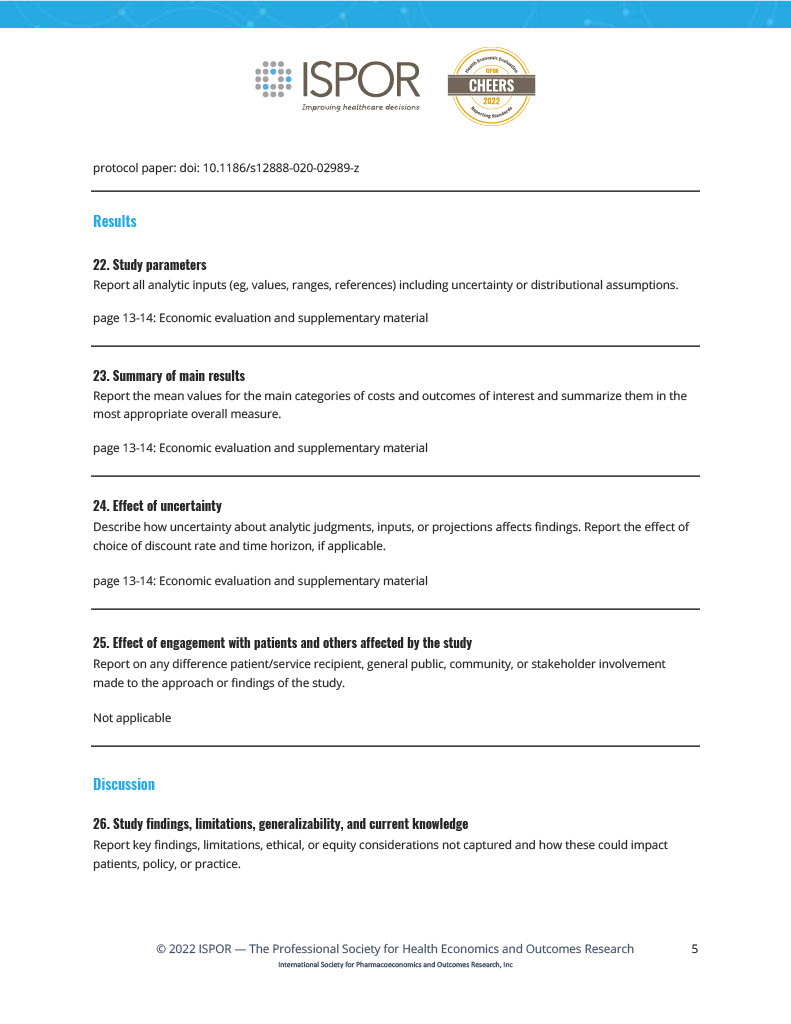
**

**
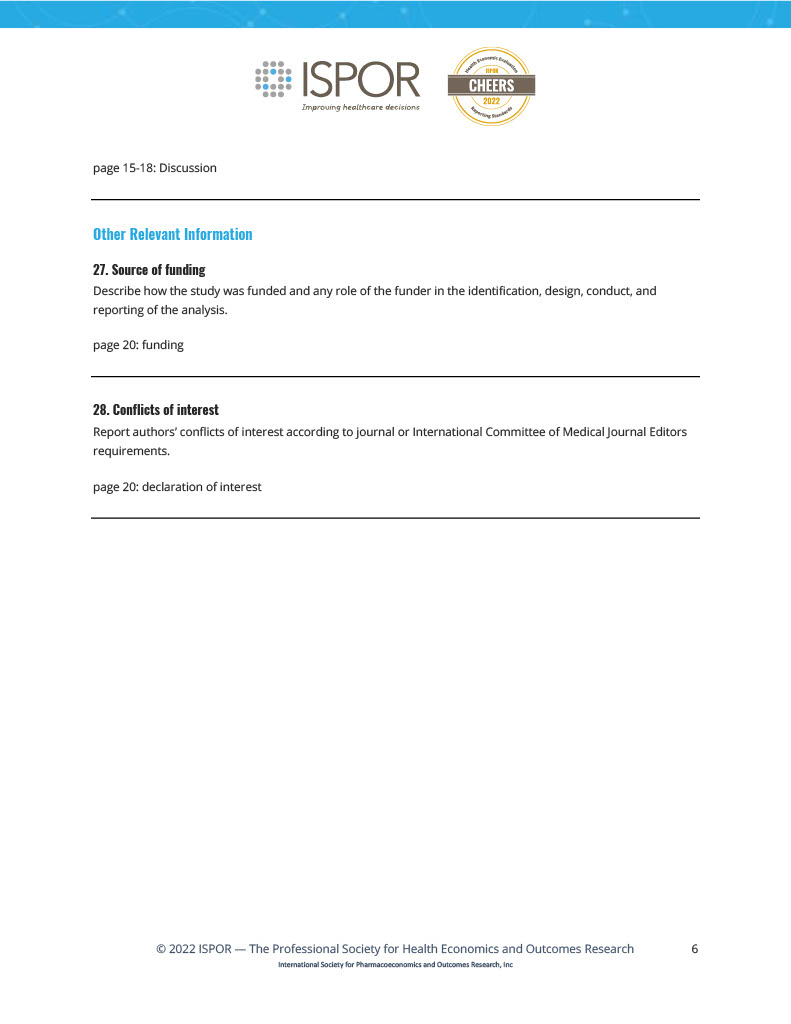
**

**References**

Basso, J. C., & Suzuki, W. A. (2017). The effects of acute exercise on mood, cognition, neurophysiology, and neurochemical pathways: A review. *Brain Plasticity*, *2*(2), 127–152. https://doi.org/10.3233/BPL-160040

Derry, P. A., & Kuiper, N. A. (1981). Schematic processing and self-reference in clinical depression. *Journal of Abnormal Psychology*, *90*(4), 286–297. https://doi.org/10.1037/0021-843X.90.4.286

Fehne, S. E, McLeod, L. D, Edin, H. M. & Hogue SL. (2004). *Development and preliminary psychometric evaluation of the motivation and energy inventory – short form (MEI-SF)*.

Franck, E., De Raedt, R., Barbez, C., & Rosseel, Y. (2008). Psychometric Properties of the Dutch Rosenberg Self-Esteem Scale. *Psychologica Belgica*, *48*(1), 25. https://doi.org/10.5334/pb-48-1-25

Gómez–Déniz, E., Gallardo, D. I., & Gómez, H. W. (2020). Quasi-binomial zero-inflated regression model suitable for variables with bounded support. *Journal of Applied Statistics*, *47*(12). https://doi.org/10.1080/02664763.2019.1707517

Twisk, J. A., Bosman, L. A., Hoekstra, T. B., Rijnhart, J. A., Welten, M. A., & Heymans, M. (2018). Different ways to estimate treatment effects in randomised controlled trials. *Contemporary Clinical Trials Communications*, *10*, 80–85. https://doi.org/10.1016/j.conctc.2018.03.008

Kambeitz-Ilankovic, L., Rzayeva, U., Völkel, L., Wenzel, J., Weiske, J., Jessen, F., Reininghaus, U., Uhlhaas, P. J., Alvarez-Jimenez, M., & Kambeitz, J. (2022). A systematic review of digital and face-to-face cognitive behavioral therapy for depression. *Npj Digital Medicine*, *5*(1), 144. https://doi.org/10.1038/s41746-022-00677-8

Raes, F. , Hermans, D. , & Eelen, P. (2003). *De Nederlandstalige versie van de Ruminative Response Scale (RRS-NL) en de Rumination on Sadness Scale (RSS-NL) [The Dutch version of the Ruminative Response Scale (RRS-NL) and the Rumination on Sadness Scale (RSS-NL)]*.

Schmitter, M., Spijker, J., Smit, F., Tendolkar, I., Derksen, A.-M., Oostelbos, P., Wijnen, B. F. M., van Doesum, T. J., Smits, J. A. J., & Vrijsen, J. N. (2020). Exercise enhances: study protocol of a randomized controlled trial on aerobic exercise as depression treatment augmentation. *BMC Psychiatry*, *20*(1), 585. https://doi.org/10.1186/s12888-020-02989-z

Ustun, Tevfik Bedirhan, Kostanjesek, N, Chatterji, S, Rehm, J. & W. H. O. (2010). *Measuring health and disability : manual for WHO Disability Assessment Schedule (‎WHODAS 2.0)*. Retrieved from: https://apps.who.int/iris/handle/10665/43974

Vandelanotte, C., De Bourdeaudhuij, I., Philippaerts, R., Sjöström, M., & Sallis, J. (2005). Reliability and validity of a computerized and Dutch version of the international physical activity questionnaire (IPAQ). *Journal of Physical Activity and Health*, *2*(1), 63–75. https://doi.org/10.1123/jpah.2.1.63

Vijgen, S. , van H. F. , & O. M. (2018). Ziektelast in de praktijk; De theorie en praktijk van het berekenen van ziektelast bij pakketbeoordelingen [Disease burden in practice: The theory and practice of calculating disease burden in package evaluations].

Wardenaar, K. J., van Veen, T., Giltay, E. J., den Hollander-Gijsman, M., Penninx, B. W. J. H., & Zitman, F. G. (2010). The structure and dimensionality of the Inventory of Depressive Symptomatology Self Report (IDS-SR) in patients with depressive disorders and healthy controls. *Journal of Affective Disorders*, *125*(1–3), 146–154. https://doi.org/10.1016/j.jad.2009.12.020
